# Supplementary figures and images for: Reduction of endocytosis and EGFR signaling is associated with the switch from isolated to clustered apoptosis during epithelial tissue remodeling in Drosophila
Source: PLoS Biol. 2024 Oct 14;22(10):e3002823. doi: 10.1371/journal.pbio.3002823 (PMC11472926; doi:10.1371/journal.pbio.3002823)

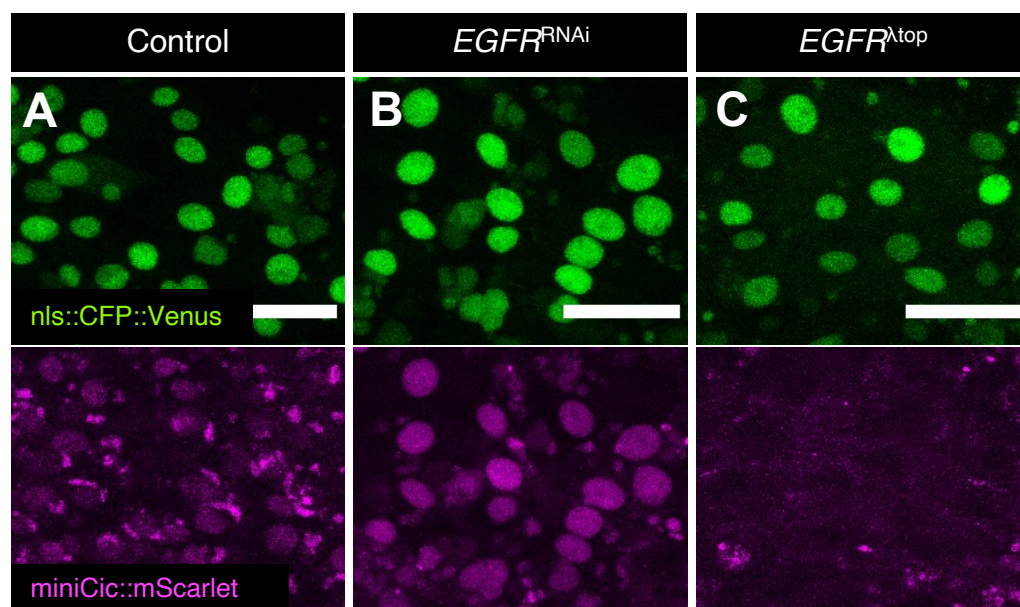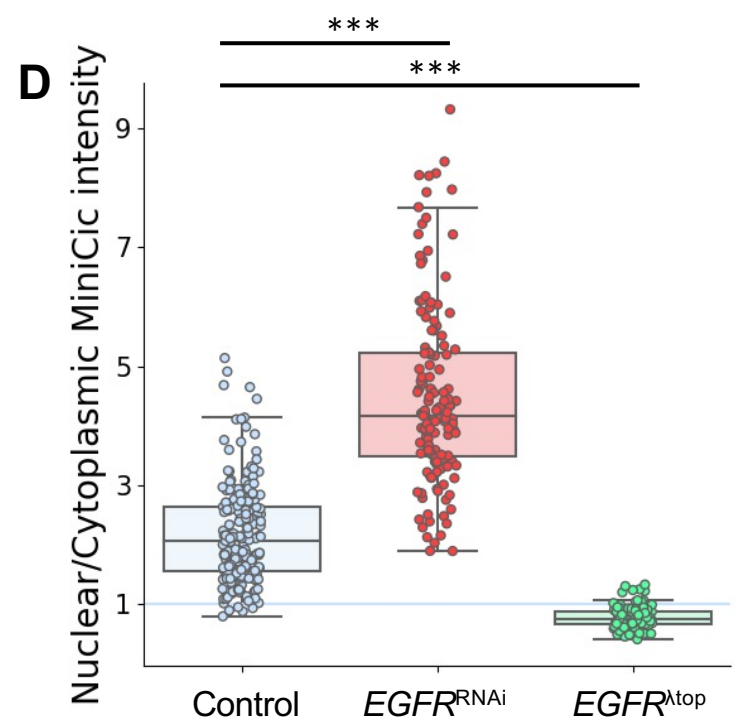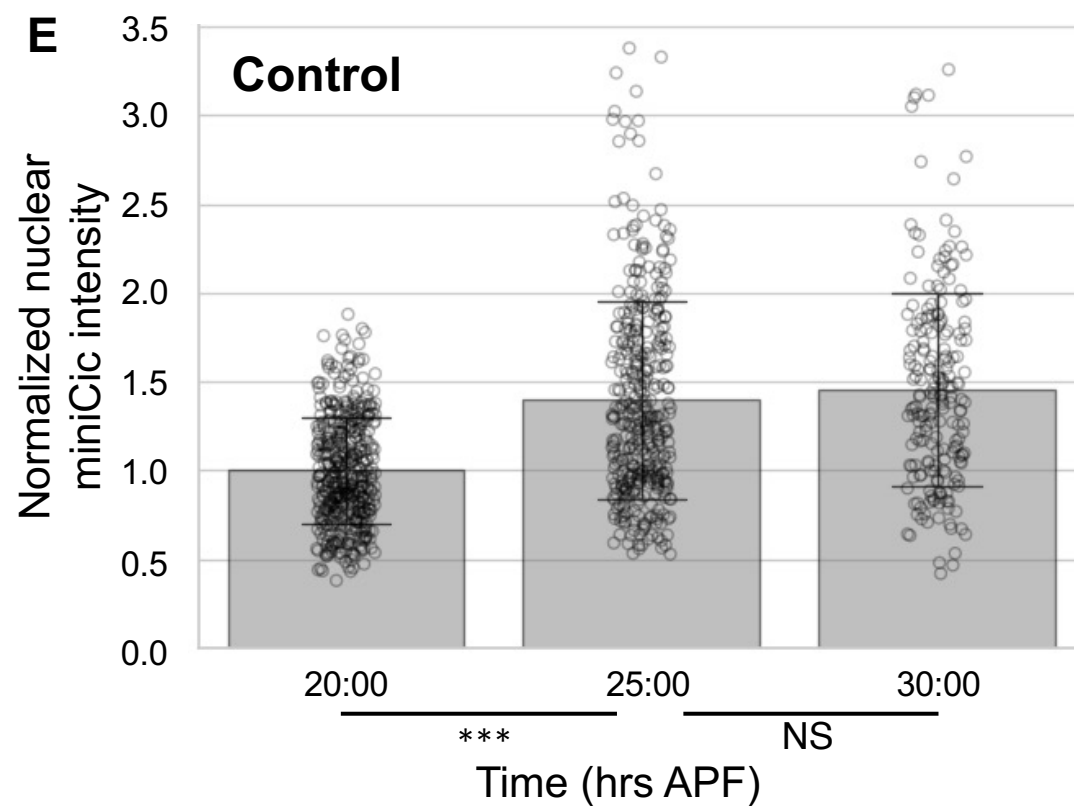

Fig. S1

Supplement: S1 Fig — (A–C) Top row, nuclear signals and bottom row, miniCic signals in LECs at 20 hAPF in (A) control, (B) EGFRRNAi, and (C) EGFRλtop expressing pupae. Scale bars: 50 μm. (D) Nuclear / cytoplasmic ratio of miniCic in LECs at 20 hAPF. n = 50 cells / pupa, 3 pupae. (E) Normalized nuclear miniCic intensity in control LECs at 20, 25, and 30 hAPF. Error bars are SEM Mann–Whitney test, ***P < 0.001. Genotypes: (A) ywhsFlp/+; tubP-miniCic::mScarlet/+; UAS-nls::CFP::Venus, pnr-GAL4/+. (B) ywhsFlp/+; tubP-miniCic::mScarlet/+; UAS-nls::CFP::Venus, pnr-GAL4/ UAS-EGFR RNAi. (C) ywhsFlp/+; tubP-miniCic::mScarlet/+; UAS-nls::CFP::Venus, pnr-GAL4/ UAS-EGFR λtop. The data underlying the graphs shown in the figure can be found in https://zenodo.org/records/13290047. (PDF) [file pbio.3002823.s001.pdf]

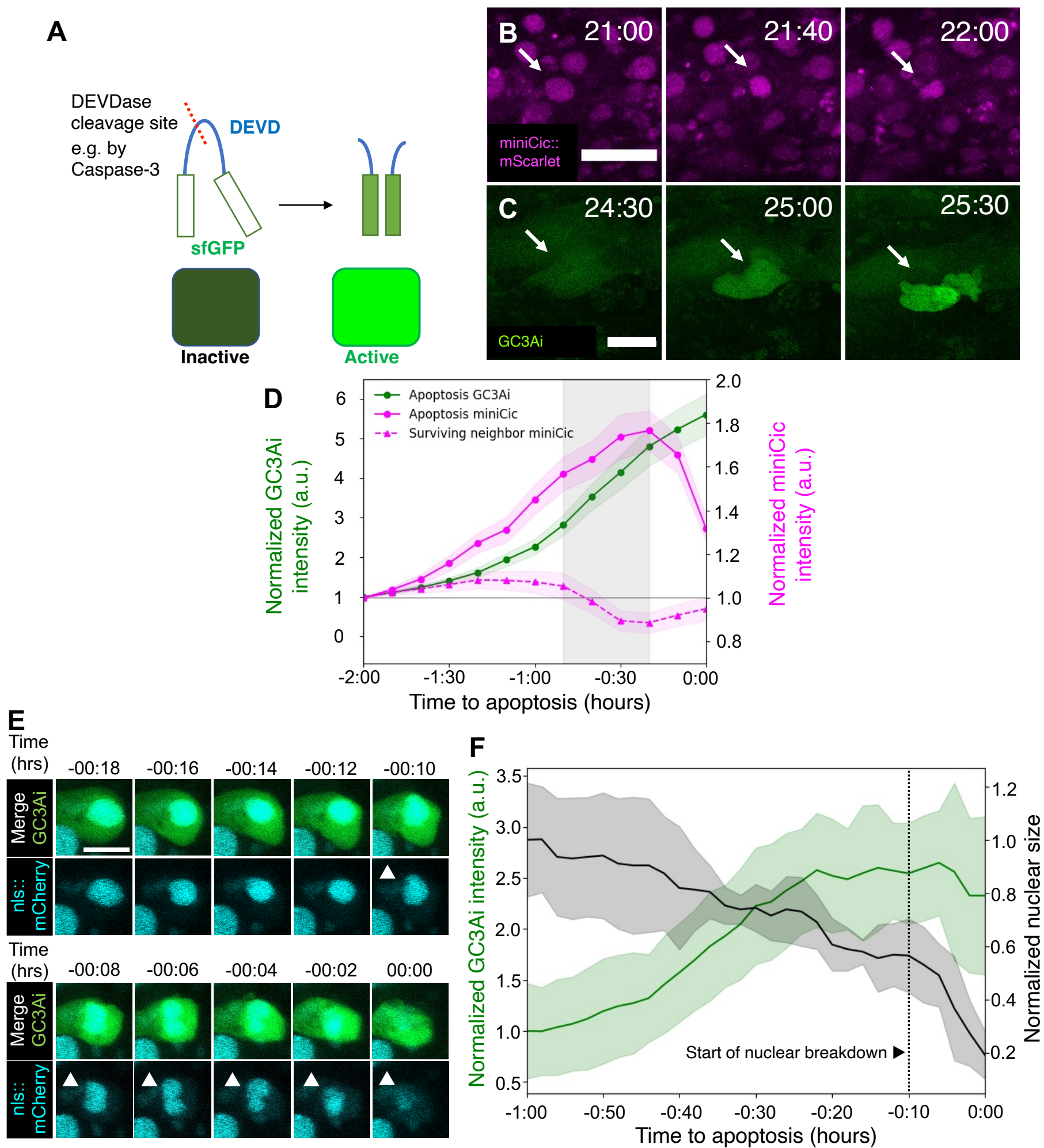

Fig. S2

Supplement: S2 Fig — (A) Schematic of GC3Ai reporter of apoptotic caspase activity. (B) miniCic signals and (C) caspase activity in LECs in control pupae. White arrows indicate apoptotic LECs. Time indicates hours APF. Scale bars: (B) 50 μm and (C) 20 μm. (D) Plot of the time scale of normalized miniCic and GC3Ai intensity in LECs, aligned to the time of cell death. n = 10 cells / pupa, 3 pupae. (E) Confocal images of nls::mCherry and GC3Ai in an apoptotic LEC. Time indicates hours to apoptosis. White arrowhead indicates ongoing nuclear breakdown. (F) Plot of the time scale of normalized GC3Ai intensity and nuclear sizes in LECs, aligned to the time of cell death. n = 2–3 cells / pupa, 3 pupae. Scale bar: 20 μm. Errors are SEM. Genotypes: (B) ywhsFlp/+; tubP-miniCic::mScarlet/+; UAS-nls::CFP::Venus, pnr-GAL4/+. (C) ywhsFlp/+; +/+; pnr-GAL4, UAS-GC3Ai/+. (E) ywhsFlp/+; UAS-nls::mCherry/+; pnr-GAL4, UAS-GC3Ai/+. (E, F) ywhsFlp/+; UAS-GC3Ai/+; pnr-GAL4/UAS-nls::mCherry. The data underlying the graphs shown in the figure can be found in https://zenodo.org/records/13290047. (PDF) [file pbio.3002823.s002.pdf]

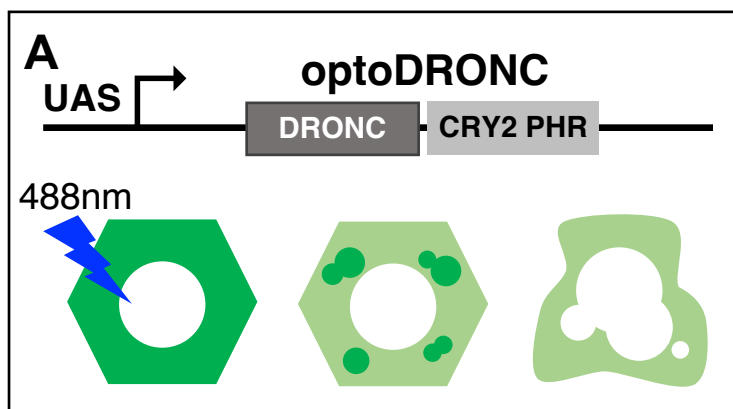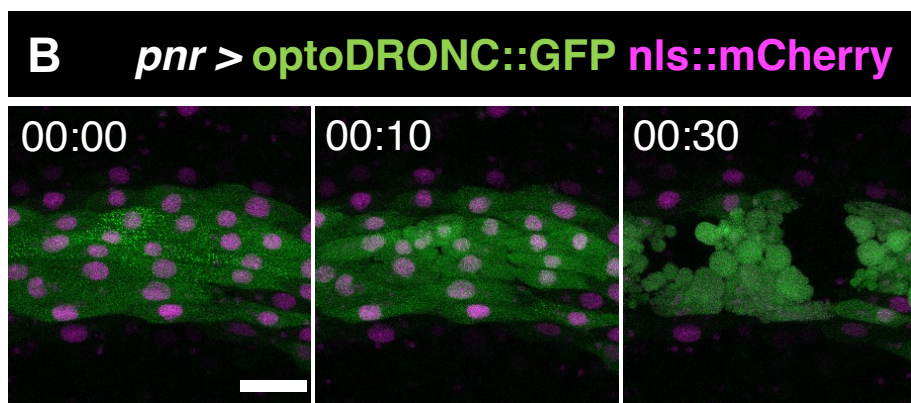

Fig. S3

Supplement: S3 Fig — (A) Schematic of OptoDRONC (top) and workflow for optogenetically inducing OptoDRONC (bottom). (B) General exposure to 488 nm laser to activate OptoDRONC in all pnr-expressing LECs promotes death. Time indicates hours after 488 nm laser exposure. Scale bar: 50 μm. Genotypes: (B) ywhsFlp/+; UAS-OptoDRONC::GFP/+; UAS-nls::mCherry, pnr-GAL4/+. (PDF) [file pbio.3002823.s003.pdf]

## A Cluster death analysis and *in silico* simulation workflow

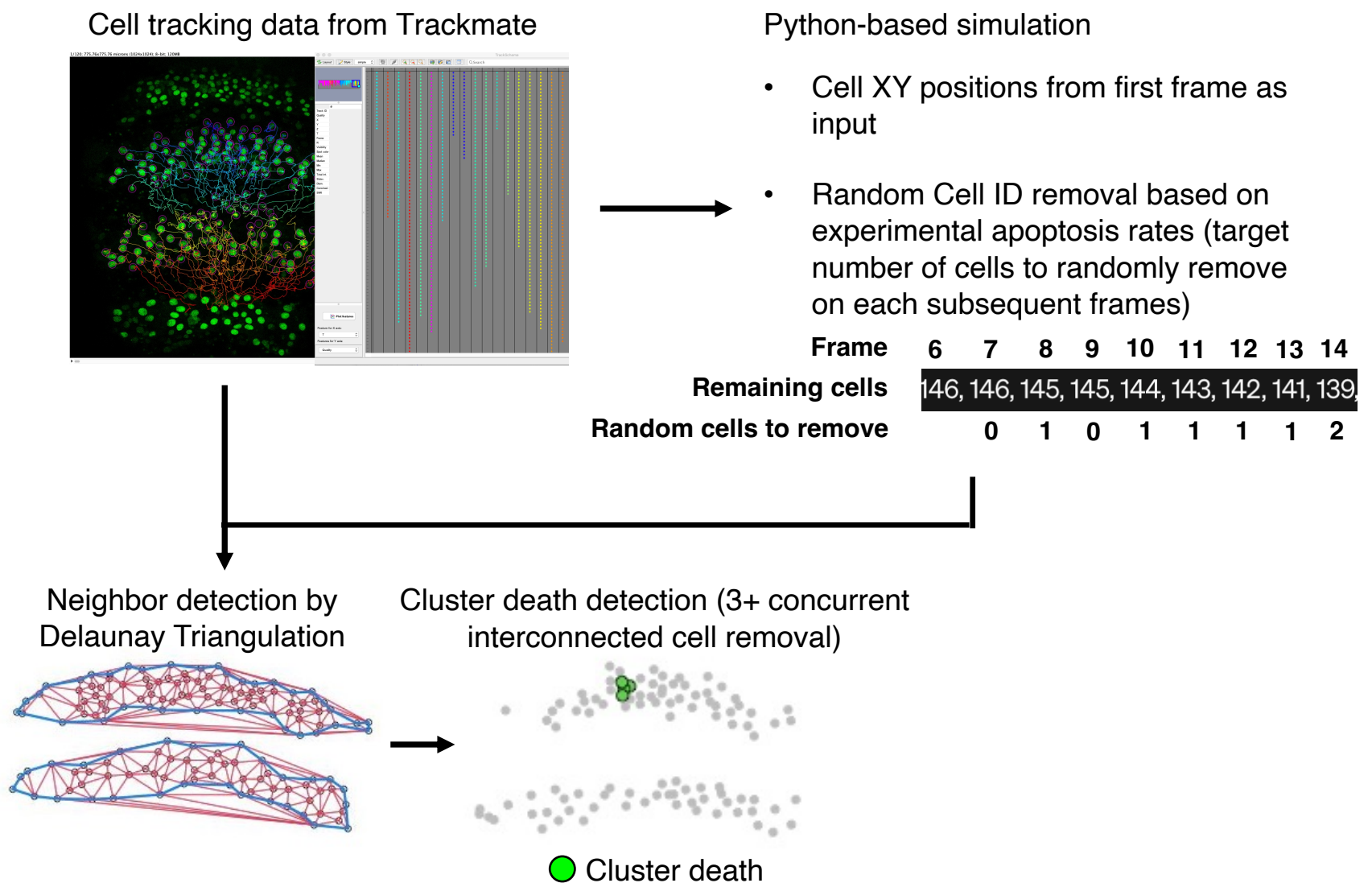

## B

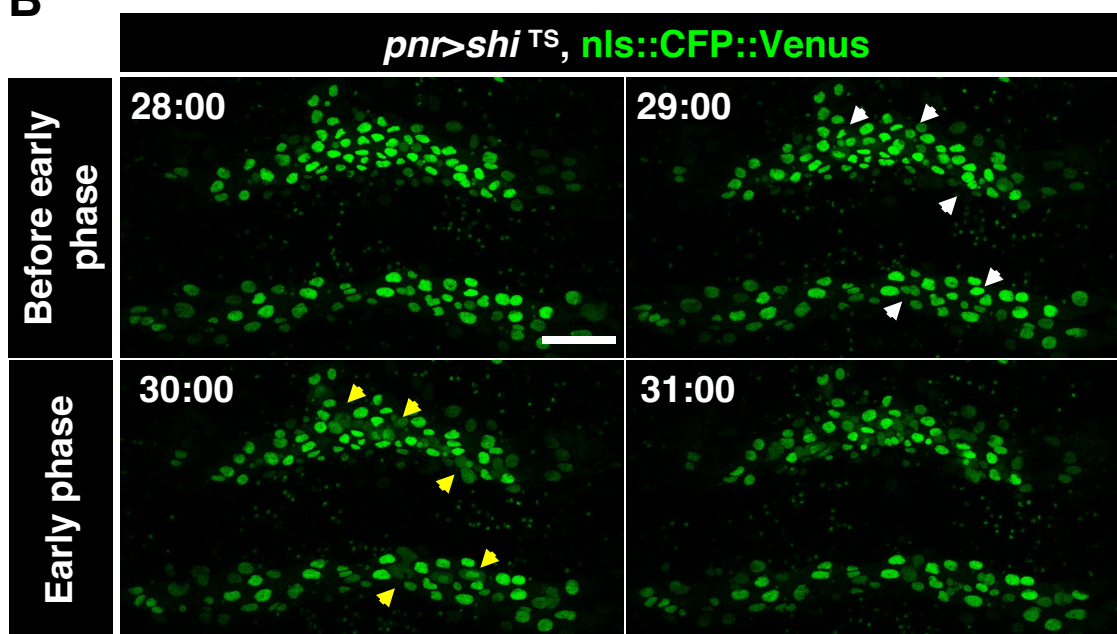

Supplement: S4 Fig — (A) Workflow of cluster cell death detection and in silico simulation for randomized LEC apoptosis. Cell tracking data from TrackMate are set as the input for LEC neighbor detection via Delaunay Triangulation. Pairs dying concurrently are labeled, and if two of more pairs are interconnected, they are determined as cluster deaths. (B) Confocal imaging of nls::CFP::Venus of shi TS expressing LECs starting from 28 hAPF, 2 h before early phase at 30 hAPF. The first signs of clusters of cells preparing for apoptosis starts at 29 hAPF (white arrows) which completes with nuclear breakdown at 30 hAPF (yellow arrows). Scale bar: 100 μm. Genotypes: (B) ywhsFlp/+; tubP-miniCic::mScarlet/+; UAS-nls::CFP::Venus, pnr-GAL4/ UAS-shi TS. The data underlying the graphs shown in the figure can be found in https://zenodo.org/records/13290047. (PDF) [file pbio.3002823.s004.pdf]

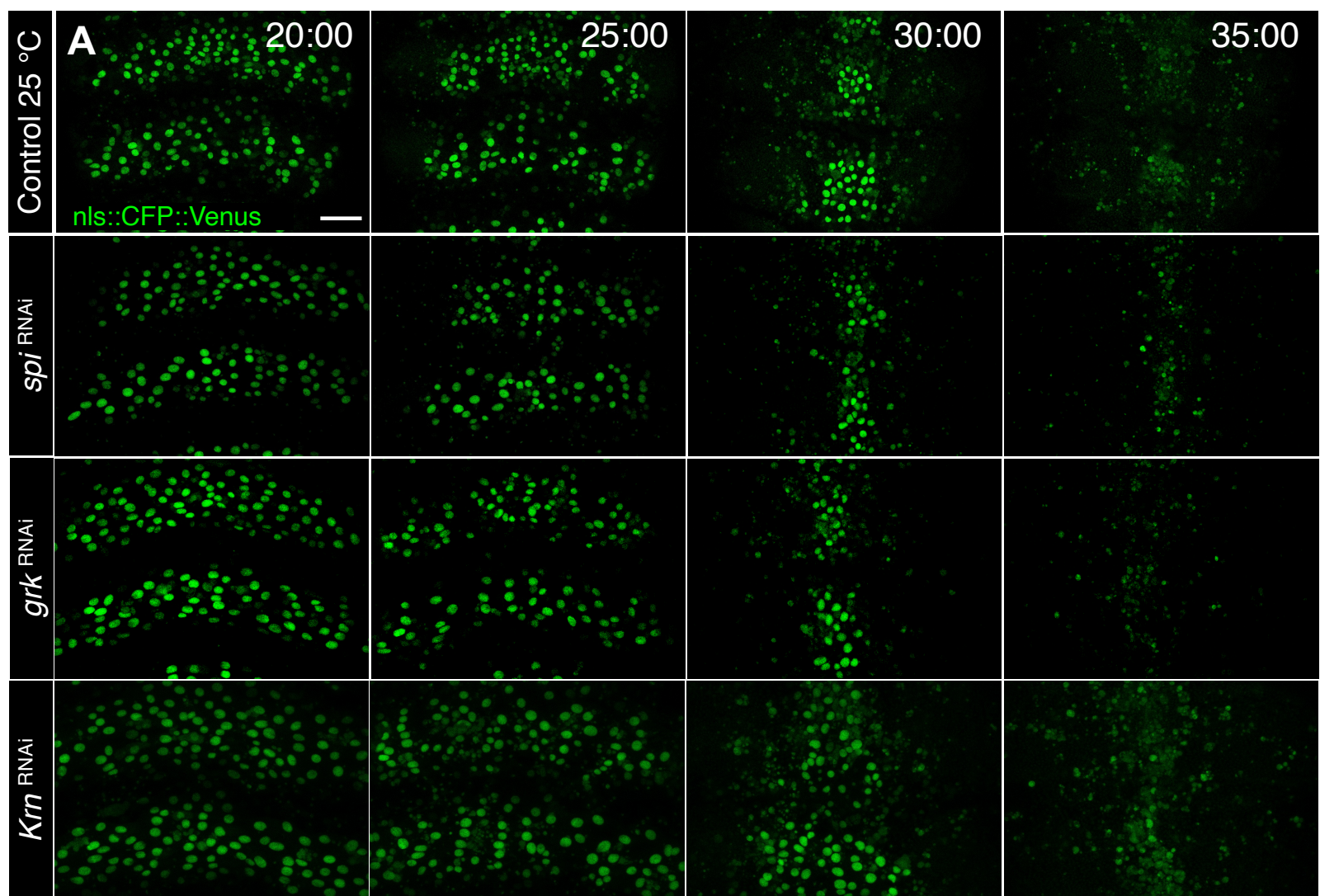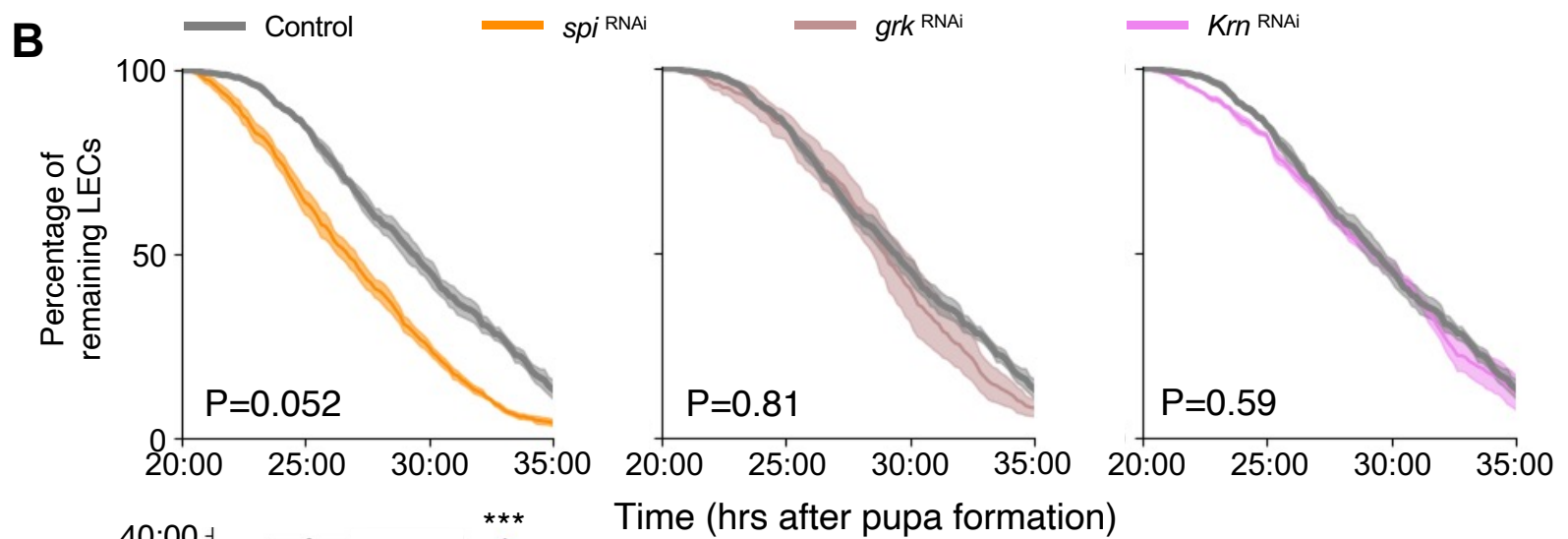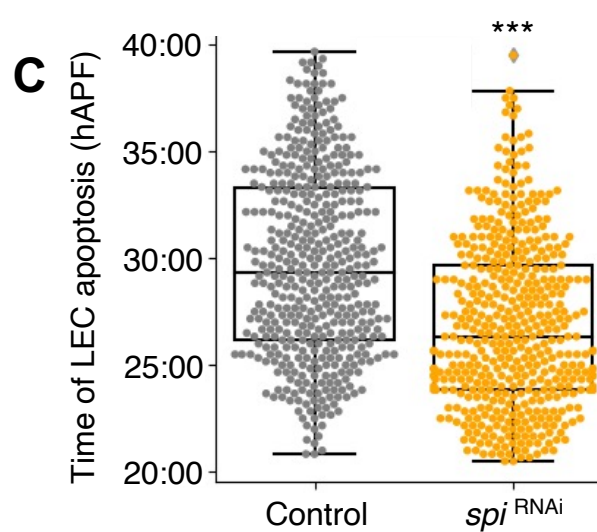

Fig. S6

Supplement: S6 Fig — (A) nls::SCAT3 expression in, from top to bottom: control, spi RNAi, grk RNAi, and Krn RNAi pupae. Time indicates hours APF. Scale bars 50 μm. (B) Percentage of remaining LECs in control vs. from left to right: spi RNAi, grk RNAi, and Krn RNAi pupae. n = 3 pupae each. Error bars are SEM. Kolmogorov–Smirnov test vs. control. P-values are as indicated. (C) Swarmplot of LEC apoptosis counts at each time point for control vs. spi RNAi LECs. Each dot represents 1 cell. n = 3–4 pupae. Mann–Whitney test vs. Control. ***P < 0.001. Genotypes: (A–C) ywhsFlp/+; UAS-nls::CFP::Venus/+; pnr-GAL4/ +. ywhsFlp/+; UAS-nls::CFP::Venus/+; pnr-GAL4/ UAS-spi RNAi. ywhsFlp/+; UAS-nls::CFP::Venus/+; pnr-GAL4/ UAS-grk RNAi. ywhsFlp/+; UAS-nls::CFP::Venus/+; pnr-GAL4/ UAS-Krn RNAi. (PDF) [file pbio.3002823.s006.pdf]
