# Supplementary material for: Reduction of endocytosis and EGFR signaling is associated with the switch from isolated to clustered apoptosis during epithelial tissue remodeling in Drosophila
Source: PLoS Biol. 2024 Oct 14;22(10):e3002823. doi: 10.1371/journal.pbio.3002823 (PMC11472926; doi:10.1371/journal.pbio.3002823)
Supplement: S5 Fig — (A) Representative confocal images and diagrams showing various shapes and sizes of clustered LEC apoptosis. Time stamp indicates time to apoptosis in hours. Scale bars 50 μm. (B) Boxplot of the number of cells in single LEC clustered apoptosis events between control, EGFR RNAi, and shi TS pupae. Error bars are SEM. Mann–Whitney test, ***P < 0.001. (C) Confocal images of a cluster of apoptotic EGFR RNAi expressing LECs represented using cell outlines (neuroglian::GFP, NRG::GFP). Clustered apoptosis is outlined by cyan lines. White arrows indicate epithelial sealing upon the completion of clustered apoptosis. Yellow arrowheads indicate an expanding nearby histoblast nest. Time stamp indicates time to apoptosis in hours. Scale bar 50 μm. Genotypes: (A, B) ywhsFlp/+; tubP-miniCic::mScarlet /+; UAS-nls::CFP::Venus, pnr-GAL4/+. ywhsFlp/+; tubP-miniCic::mScarlet /+; UAS-nls::CFP::Venus, pnr-GAL4/ UAS-EGFRRNAi. ywhsFlp/+; tubP-miniCic::mScarlet /+; UAS-nls::CFP::Venus, pnr-GAL4/ UAS-shiTS. (C) ywhsFlp/+; NRG::GFP/+; pnr-GAL4/ UAS-EGFRRNAi. The data underlying the graphs shown in the figure can be found in https://zenodo.org/records/13290047. (PDF) [file pbio.3002823.s005.pdf]

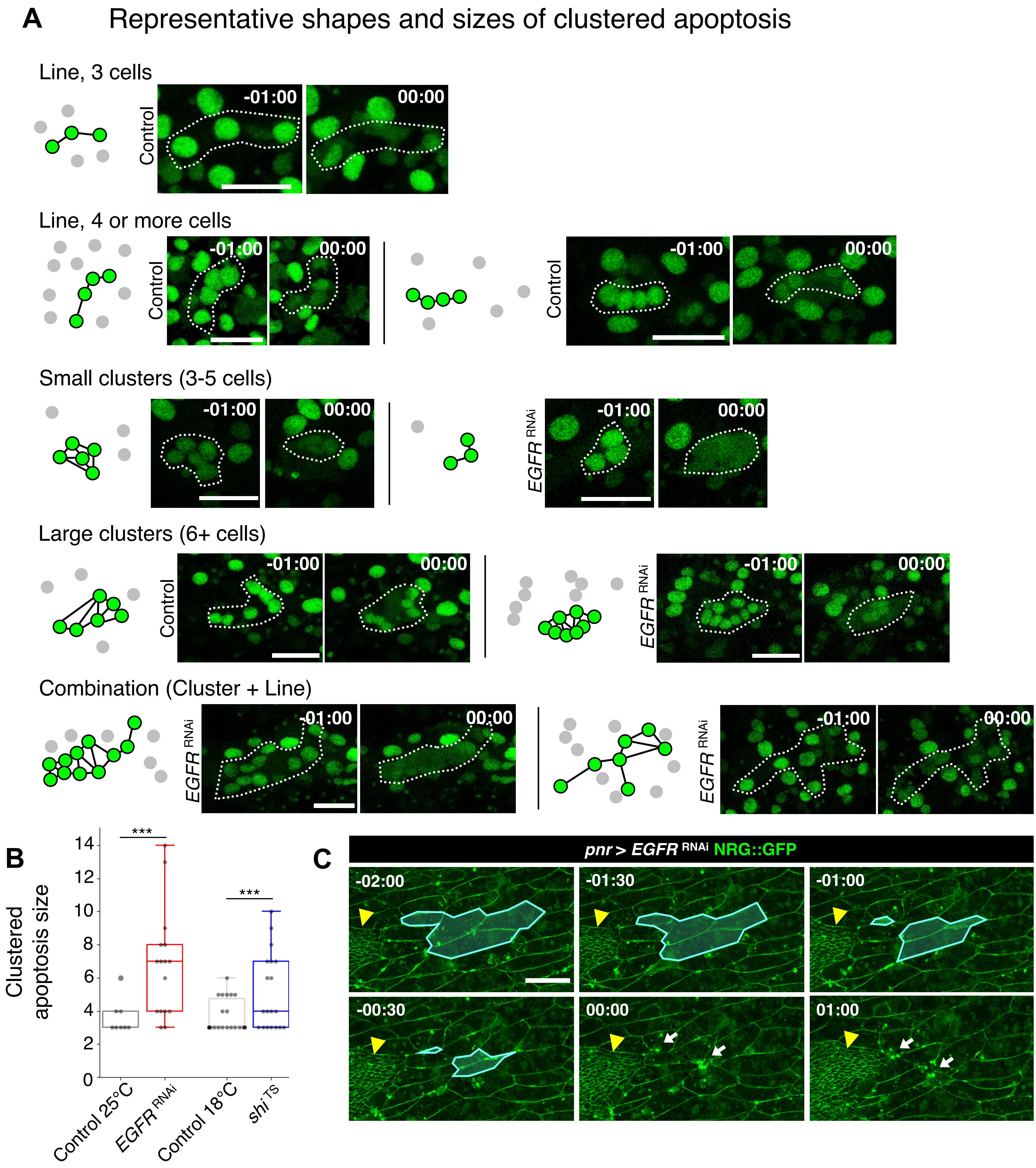

A
Representative shapes and sizes of clustered apoptosis
Line, 3 cells
Control
-01:00
00:00
Line, 4 or more cells
Small clusters (3-5 cells)
EGFR RNAi
Large clusters (6+ cells)
Combination (Cluster + Line)
B
Clustered apoptosis size
***
14
12
10
8
6
4
2
0
Control 25°C
EGFR RNAi
Control 18°C
shi TS
C
pnr > EGFR RNAi NRG::GFP
-02:00
-01:30
-01:00
-00:30
00:00
01:00

Fig. S5
