# Supplementary material for: Reduction of endocytosis and EGFR signaling is associated with the switch from isolated to clustered apoptosis during epithelial tissue remodeling in Drosophila
Source: PLoS Biol. 2024 Oct 14;22(10):e3002823. doi: 10.1371/journal.pbio.3002823 (PMC11472926; doi:10.1371/journal.pbio.3002823)
Supplement: Attachment — Submitted filename: EGFR_rebuttal submitted.docx [file pbio.3002823.s013.docx]

Reviewers' responses

**We would like to thank all the reviewers for overall positive and constructive comments. We have revised our manuscript based on their comments. We wrote our response in a point-by-point manner as follows and highlighted modified/added statements with red characters in the main text.**

Rev. 1:
In this study, Yuswan and colleagues characterised an interesting development switch which through the downregulation of endocytosis reduces EGFR/ERK activity, the occurrence of ERK pulses near dying cells and as such promotes the formation of clusters of cell elimination and accelerate the elimination of larval epidermal cells. This work is a follow-up from a previous study from the same authors which showed a global reduction of endocytosis levels during late pupal development, which was responsible for an acceleration of cell elimination. Moreover, this study recapitulates to a large extend a previous study performed in the pupal notum showing the existence of EGFR/ERK pulses near extruding/dying cells which inhibit cell death and prevent the formation of clusters of elimination. This work nicely complement these two previously published work by two main additions : 1) showing the role of endocytosis for the fluctuations of EGFR/ERK as well as the global downregulation of EGFR/ERK activity 2) showing for the first time the existence of a developmental switch that blocks the EGFR/ERK pulses near dying cells which as a consequence accelerate LECs elimination and allow the formation of clusters of cell elimination.

The study is well performed, with all the adequate control and quantifications (apart from few details for quantification and putative limitations, see below). It is also good to see that some of the process observed in the pupal notum can be recapitulated in the LECS to a great extent. Of note, the existence of EGFR/ERK pulses near extruding larval epidermal cell of the abdomen was already shown in Valon et al Dev Cell 2021 (Figure S4). It does not remove any novelty about the two main points I mention above, but I think it would be fair to mention it somewhere in the main text. I am overall very supportive for publication in Plos Biology as it opens very interesting perspective on physiological switch of apoptosis rate related to endocytosis and ERK dynamics, which may be relevant in other contexts/ tissues. Given the relative ubiquitous observation of ERK pulses and waves near dying cells (MDCK cells, MCF10A cells, HelA cells, and more recently human colon organoid),
these results might be of very broad interest.

I still have though some important suggestions requiring text and figure editing, to match what can be really conclude firmly with the current data. I also have some suggestions to clarify some results and quantifications.

**Our response**
**We thank Reviewer 1 for a lot of constructive comments and showing the great interest in our findings. Please find below our response to each specific comment.**

Main points : Poisson analysis, Rab5 ERK pulses
1/ The authors made strong statements about the occurrence of clusters of cell elimination and their direct role for the acceleration of LECs elimination rate. While it is true that their data show a striking correlation between the two, it is actually pretty hard to distinguish what is the effect of a global increase of the rate of cell elimination (irrespective of its spatial distribution) and what is the contribution a specific spatial distribution of cell death. Unless that authors have a way to compare two conditions where global death rate is the same, but where the clustering is different, I would maybe suggest to clearly describe this limitation and tune down the statements in the title, abstract , results and discussion (and I don't think this will reduce the interest of this study). For instance, the short title "Clustered apoptosis regulates tissue remodeling rate" looks like a strong statement and I would rather use something more neutral like "A switch to
cluster apoptosis is associated with accelerated tissue remodeling".

**Our response**

**We appreciate the critical comment about detailing the spatial patterns of apoptosis with the global rate of LEC elimination. We also had a few concerns about whether the increased rate of LEC elimination will increase the chances of clustered apoptosis in the end (related to comment #3). To address these issues, we have performed simple simulations in which cells are eliminated in a stochastic manner (Poisson process) starting from the configuration of cells found in our experiments (see Fig. 6F, S4, and Materials and Methods). We used the LEC elimination rate from the experimental data and randomized the selection of cells to remove at each time point. We found that clustered eliminations are extremely rare from our multiple iterations of each sample compared to the *in vivo* observations, even simulating the high elimination rate of LECs expressing EGFR RNAi and *shi* ^TS^. Therefore, the model supports our idea that clustered elimination is not a mere consequence of high elimination rate and increased fraction of dying cells. Concerning the overstatements that the reviewer pointed out, we agree with the reviewer that some statements are too strong. However, since our simulations, which showed that the cluster cell elimination is not a mere consequence of a frequent random cell elimination, gave an additional support for our finding as the original title saying “Switching to clustered apoptosis induced by reduction of endocytosis and EGFR signaling accelerates Drosophila tissue remodeling”, we think that the title can stay as it was. The other parts including short title, abstract, results, and discussion were tuned down to convey more neutral nuance as shown below.**

**Short title: “Tissue remodeling-associated clustered apoptosis”**

**Abstract (Line 43): “… the acceleration is accompanied by a mode switching….”**

**Results (Line 396-400): “… and the loss of transient ERK upregulation induces clustered LEC apoptosis, which is associated with the acceleration of cell elimination rate….”**

**Discussion (Line 472-475): “…suggesting that the acceleration of cell elimination in the later stage is linked to the switch from single-cell to clustered-cell apoptosis.”**

2/ Along the same line, this is currently difficult to distinguish what is the relative impact of ERK pulses near dying cells versus the global modulation of ERK basal activity. Both could equally contribute to an increase rate of cell elimination and as far as I can judge, the authors don't really have a condition that can sort these two parameters. I would therefore suggest to mention clearly this limitation in the discussion

**Our response**

**We agree with the concern about this issue. As the reviewer pointed out, we currently do not have a condition to modulate the two parameters independently. This is technically challenging since we do not know the regulatory mechanism for the down-regulation of the global ERK basal activity and is actually the limitation of this study. We have added the relevant discussion and statements into the Discussion section in lines 540-550:**

**“A limitation of this study is in fact that there are no conditions to independently modulate and uncouple the basal ERK activity and the ERK pulses. While the global ERK downregulation seems to reflect global downregulation of endocytic activity in LECs, it is still unclear if the global downregulation also contributes to the switch in cell elimination mode. One possibility of the role of global downregulation of ERK basal activity is that it may desensitize LECs to the transient ERK pulses and ensure cluster cell elimination occurs during later stages. The ERK pulses were reported to depend on frequency and intensity thresholds to successfully induce apoptosis-induced-survival [38]. In this way, despite the continuous presence of ERK pulses, the low basal ERK activity together with the reduced levels of ERK pulses in late phase LECs cause the total ERK activity unable to reach the required survival thresholds.”**

**We have also added another potential mechanism in the discussion that Vn, and to a lesser degree Spi have a global function which is permissive and required to prevent clustered apoptosis in the early phase which is independent of the ERK pulses. In this way, the increase of clustered apoptosis may be due to the global downregulation of ERK caused by several possibilities: (1) reduced ligand availability, or the reduction of endocytic activity which leads to either (2) lower EGFR/ERK pathway downstream activation despite ligand binding, as proposed in the Discussion section. We have added the relevant discussion and statements regarding this issue into the Discussion section as provided in the lines 580-586.**

**“Another potential mechanism is that Vn and to a minor degree Spi, have a global permissive function where the presence of the ligand is prerequisite for preventing clustered cell elimination in the early phase independent of the regulation of the transient ERK activation. In this case, either: (1) the reduced ligand availability, or (2) the loss of EGFR availability and ligand-dependent activation due to the loss of endocytic activity can cause the global downregulation of the ERK signal to the level that cannot be overcome anymore by pulsed upregulation of the ERK for cell survival.”**

3/ So far, the authors interpret the increase occurrence of clusters by the reduction of ERK pulses near dying cells, however if everything being random ( a pure Poisson process), the probability to observe cluster will also increase if the global rate of cell elimination is higher (independently of any local feedback), and could thus be explained by the change of the basal levels of ERK activity (see point 2). Is there any condition where the authors could see relatively mild effect on the global speed of elimination and yet very clear increase of the number of clusters ? That could help to argue for a specific effect on cell death distribution. Alternatively, one would need to go for a much more thorough description of the spatiotemporal distribution of cell death to prove that there is different "clustering" levels relative to a Poisson distribution with the same rate. Again, I don't think this is absolutely necessary here (and this was already performed in other context
before) but I would as such remain cautious on this. Still, this could also be complemented by plotting the number of clusters in the different genetic background like in Figure 6I, but this time as a function of the global rate of cell elimination. That would help to partially clarify this.

**Our response**

**We appreciate the discussion about whether the higher probability of the increased LEC elimination rate can also increase the occurrence of clustered elimination. We have tried to address this issue in our response to comment #1 using the simulation to eliminate cells in a fully stochastic manner. Our results showed the very low possibility of clustered apoptosis to occur when cell removal rate is set to follow the purely randomized Poisson process. We have added the details and results of this simulation to the main text (lines 381-392), Fig. 6, S4, and the Materials and Methods sections.**

**Lines 379-390 “These observations led us to hypothesize that clustered apoptosis is a unique mode of rapid cell removal, spatiotemporally regulated by the modulation of endocytic activity and ERK signaling. However, it is still possible that clustered elimination is a mere consequence of high elimination rate and increased fraction of dying cells in the late phase. Therefore, we compared the frequency of clustered cell elimination in tissues and that of random processes by implementing *in-silico* simulations. The simulations were performed using the initial cell positions and elimination rate from our experimental control, *EGFR* ^RNAi^, and *shi* ^TS^ pupae, in fully randomized spatial patterns of cell elimination (Fig. 6F, see Materials and Methods). These simulations revealed that fully stochastic order of apoptosis, despite having the elimination rates mimicking the *in-vivo* experiments, hardly yields clustered elimination (Fig. 6F). These results support our hypothesis that clustered apoptosis events are unlikely to be caused by random chances.”**

**Additionally, we added further discussion to our statement “We revealed that this tightly regulated acceleration of cell elimination rate is not a mere consequence of the increased frequency of isolated single-cell apoptosis, but rather results from a switch in the mode of cell elimination toward simultaneous apoptosis of cells in local clusters.” in lines 520-522 to emphasize this simulation results.**

**“The comparison between experimental data and simulations clearly showed that the clustered cell elimination is also not a mere consequence of the increased cell elimination rate.”**

**For the second point, we are afraid to say if we understood correctly what the reviewer suggested, but we think that the global rate of cell elimination would correlate with the number of clusters since the global cell elimination rate is accelerated by the increase of clustered cell death. Therefore, we think that the plot the reviewer suggested would not distinguish the regulated clustered cell death from the mere increase of clusters due to the increase of density of dying cells.**

Point 1,2,3 are admittedly hard to address in a very clean manner (unless one is lucky enough to find a conditions that help to disentangle these factors) and would require also quite thorough statistical characterisation of cell death distribution combined with modeling (to distinguish what is coming from a change of the absolute probability of cell death or what is driven by the direct impact of the local feedback). I don't think this change the overall interest of the study, but I think it would be fair to clearly acknowledge these limitations in the discussion and be cautious about statement along these points.

**Our response**

**We appreciate the major critical comments. As we have mentioned above, we feel that our simulations provide a strong support for our hypothesis that the clustered cell elimination is a regulated process but not just a consequence of frequent cell elimination in the late phase (see Fig. 6F, S4). In addition, we have also described some technical limitations in our simulations in the text (Lines 522-525)**

“**Further improvements in our simulations may provide even better comparisons since our simulations do not consider the midline migration and other passive movements of cells due to apoptotic events which may influence the distribution of apoptosis.** ”

**And in the Materials and Methods (Lines 686-688)**

**“While our simulation model did not incorporate the LEC migration, the triangulation finds the shortest connections between points, which may mitigate the spatial limitations.”**

4/ Along the 3 points above, did the author look at the ERK pulses in the context of active Rab5 overexpression ? If ERK pulses are affected, while global ERK activity is higher, it would be an interesting case to discuss the point above.

**Our response**

**We have analyzed the ERK pulses in the Rab5 overexpression experiment and found that the ERK pulses are still present (Response Fig.1). Since we cannot still uncouple the occurrence of ERK pulses and the global ERK activity, we are not sure whether the increase in cell elimination rate is due to the decrease in the ERK pulses or global ERK activity. Interestingly, however, we found that the occurrence of the ERK pulses started later, which corresponds with the delayed initiation of LEC elimination. This result supports our idea that ERK pulses to the neighboring cells only occur when there are apoptotic events.**

Cell IDs

20

25

30

35

Time (hrs APF)

Normalized miniCic intensity (a.u.)

**Response Fig. 1. Heatmap of normalized miniCic intensity in *Rab5* ^Q88L^ expressing LECs**

Minor points:

1/ If I understood correctly, the optoDronc experiment was performed through a global activation of Dronc in the pannier domain. However, I guess it is difficult to sort in this case the cell autonomous effect of caspases from the non-cell autonomous effect (albeit I do agree with the argument of the authors about caspase activation being autonomously associated with ERK downregulation). Still, since the LEC cells are pretty large and pretty flat, it should be doable to activate Dronc in a single cell without using two photon excitation. Have the authors tried this type of experiment ? Admitedly, I don’t think this result is really essential for their study and there is enough data showing a clear correlation between dying cells and ERK activation in the neighbours.

**Our response**

**We understand the reviewer’s concern. We aimed to target single cells in order to recapitulate the apoptosis-induced EGFR activation in neighbor cells in an experimentally inducible manner in Fig.5. However, we found that multiple cells were dying concurrently even if we exposed a single cell with a laser (Fig. 5A). We do not know the reason for this concurrent multiple cell deaths in our exposure experiments. As shown in the table below we tried our best to search for the optimum condition of laser exposure. However, we were not able to find the condition and laser settings which we can induce single LEC death (Response Table 1). Either no cell deaths or multiple cell deaths were induced in our experiments. In the end, we settled with a relatively high laser power to induce the Dronc activation in multiple cells. In addition, we have also performed the constant global exposure experiment with low laser power (at 1% 488 nm with Zeiss LSM980) as seen in Fig. S3B. In this case, all *pnr* expressing LECs underwent concurrent apoptosis**, **in accordance with the previous reports. This is contrast with the results shown in Fig. 5A, where we targeted single cells labelled with an asterisk. The details of each setting we have attempted are listed in the Response Table 1.**

**Response Table 1. Table of laser settings used in the attempts to induce OptoDRONC via single cell exposure. The yellow highlighted row indicates the settings used to produce the results in this study.**

2/ Whenever using the miniCic sensor, the authors state (both in the main text, first time in line 162, and in graph axis of figures and legends) that this is a EGFR sensor. This is not quite correct since miniCic should be sensitive to ERK activity irrespective of the upstream activators. To be totally correct, I would suggest to change everywhere in the text/legends/figures this point and replace "EGFR levels" by "ERK activity".

**Our response**

**We have changed the details for miniCic from “EGFR sensor” to state “ERK activity”.**

3/ Unless I am mistaken, Figure S1 and Figure S4 are not referred to anywhere in the text.

**Our response**

**We thank the reviewer for careful reading. We have added the references for Figures S1 and S4 in the appropriate sections.**

4/ line 74: I am wondering if there is not a typo here. Shouldn't it be LEC number decrease (rather than increase) ?

**Our response**

**It is actually the number of the LECs undergoing cell death that increases. We have rephrased to avoid any confusion caused by this expression as follows in line 73-74.**

**“In coordination with the rapidly proliferating histoblasts, the number of apoptotic LECs increases.”**

5/ Line 217: are the authors talking about the increase of miniCic at late stage that can still be observed upon mirRHG treatment (the formulation was not perfectly clear for me) ? Also, do the authors have any idea/speculation on what could explain the global lower miniCic levels at late stage in mirRHG background ? (which cannot be explained by the absence pulses near dying cells which would go the other way around). That may suggest that low sublethal caspase activity may directly downregulate ERK.

**Our response**

**We thank the reviewer for the insightful comment. Indeed, we are claiming that there is an increase of miniCic at the late stage around 30 hAPF, which indicate the homogenous decrease of EGFR/ERK activity around this time. From our quick observation with GC3Ai capsase reporter in the background of miRHG in LECs, we still saw an increase of caspase activity. We have actually utilized this notion to address the concern about sublethal caspase activity influencing ERK. Comparing the GC3Ai reporter in the background of miRHG with the ERK movies presented in the main figure, we observed caspase activity starts to increase around 35h APF (Response Fig.2) while the ERK activity decreases already around 30 hAPF. These results show that the decrease of ERK activity still precedes the increase of caspase activity, which suggests that a sublethal caspase activity may unlikely have a direct influence on the EGFR/ERK downregulation. However, we cannot rule out the possibility that a low-level caspase activity which is below the detection by GC3Ai may downregulate ERK activity forming a feedback loop. So, we added a following statement in Results in line 240-243. “****While these data suggest that the global basal ERK activity is regulated by a yet-unknown mechanism, we cannot still rule out a possibility that a low-level caspase activity downregulates the global ERK activity.”**

**Response Fig. 2. Caspase reporter GC3Ai in miRHG expressing LECs**

**Dotted area indicates *pnr*-expressing LEC region. Scale bar: 50 μm.**

6/ The measurement of ERK fluctuations are interesting, however the way it is done right now (Imax-Imin) should be greatly influenced by the absolute levels of miniCic signal (similar to any standard deviation that tends to scale with the mean). Would the authors obtain similar results it this would be normalised by the average levels of miniCic ? In a way, this would be similar to look at a coefficient of variation.

**Our response**

**We are afraid to say that we are not fully convinced by the reviewer’s concern this case. While we agree that the fluctuations would scale with the mean in general, we think that it may not apply to our particular experiments since there is a caveat that the normalization could overestimate the fluctuation. The signals may be easily affected by a basal level fluctuation derived from noises derived from imaging or expression levels of miniCic if the average miniCic reporter signal is low. We would not consider the normalization is the best way to evaluate the actual miniCic fluctuations in this case. From these reasons we would like to have the quantification in the original shape.**

7/ For the optogenetic experiment, this is a detail, but I would maybe suggest another terminology in the text and figure ("bleach" "irradiated") that convey the wrong idea that one need very high laser power to obtain activation. Actually power similar to the one used for imaging should be sufficient to activate CRY2 (most of the optogenetic system can be activated at very low light intensity). I would suggest to use something more neutral like "blue light exposure".

**Our response**

**We appreciate the suggestions to change the terminologies in our reporting for the optogenetic results, and changed the “bleach” and “irradiated terms into “exposure”.**

8/ Figure 6I: there is a miss-match between the x axis title, the legend and the description in the main text (line 327). Are the value on x axis representing the average miniCic basal levels or are they representing the value of miniCic during pulses near dying cells ? I would suggest to clarify this point (both measurements would actually be relevant, again related to the main point 1, 2 and 3 above) .

**Our response**

**We apologize for the confusion regarding the correlation plot. The x-axis values represent the average nuclear/cytoplasmic miniCic throughout all LECs (revised Fig. 6G).**

9/ Line 440-441 : I would suggest to maybe reformulate the statement about artificial conditions. Indeed previous studies have characterised clustered elimination that appear upon perturbation of EGFR/ERK using exactly the same sort of genetic background and conditions used by the author here, so I was a bit surprised by this statement. However what is clearly new here is the "physiological" switch to clustered elimination that was never described before to my knowledge ( which is exactly what the authors point at in the next sentence). Unless the authors have a strong reason to keep this statement line 440, I would suggest to remove it (it is ambiguous and not really justified).

**Our response**

**We appreciate the valuable input for the statement. We had intended to enter the discussion about the new physiological role of cluster elimination in tissue development by stating similar phenomena in more homeostatic conditions using the same methods. However, as suggested it instead came ambiguous and unnecessary for the next sentence. We have removed this statement and modified the paragraph for clarity in lines 515-530**

**“Studies in developing tissues have focused primarily on isolated single cell delamination, which occurs stochastically [66–68]. By contrast, our study focuses on a massive cell removal process that is programmatically regulated throughout development.** **We revealed that this tightly regulated acceleration of cell elimination rate is not a mere consequence of the increased frequency of isolated single-cell apoptosis, but rather results from a switch in the mode of cell elimination toward simultaneous apoptosis of cells in local clusters. The comparison between experimental data and simulations clearly showed that the clustered cell elimination is also not a mere consequence of the increased cell elimination rate. Further improvements in our simulations may provide even better comparisons since our simulations do not consider the midline migration and other passive movements of cells due to apoptotic events which may influence the distribution of apoptosis. Our study constitutes the first example of implementing clustered cell elimination in the context of development and demonstrates that it plays an active role in accommodating the proliferating cells of neighboring tissues – in our model, the *Drosophila* adult histoblasts – which increase exponentially and thereby replace the shrinking tissue more rapidly as tissue remodeling progresses. The role of clustered elimination in this context is distinct from the conventional understanding, that clustered apoptosis is disadvantageous by compromising tissue integrity.”**

10/ Discussion : the authors describe an apparent contradiction between the requirement of a ligand and the putative role of mechanics in ERK activation. I would argue that the fact that the process is ligand dependent is not at all incompatible with a mechanical gated mechanism, specially since the authors have performed global Vein down-regulation (which might be globally permissive for ERK activation) and have not shown that this is specifically required in the dying cell. For instance, the ERK waves described during collective migration in MDCK cells are clearly mechanical dependent and yet also require several EGFR ligands (see work from Hirashima lab). I would suggest to be more nuanced on this statement and clearly mention the limitation of the experimental data regarding the role of Vein in dying cells versus a more global permissive function.

**Our response**

**We thank the reviewer for the constructive comment. We agree with the view that both ligand-dependent and the mechanical-gated mechanisms are not mutually exclusive and most likely work together in regulating the ERK pulses. Unfortunately, as the reviewer mentioned, we currently only have performed the global downregulation of *vein*, and were unable to locally manipulate *vein* expression in smaller LEC clusters. Additionally, we have observed in preliminary data that neighbor cell stretching induced by the primary apoptotic cell constriction is also present in LECs. Accordingly, we added/modified several sentences in the paragraph regarding the coordination between the ligand dependent and mechanical gated mechanisms (line 558-567).**

**“Ligand-dependency of this mechanically-gate ERK waves has been previously reported in MDCK cell collective migration [71]. Ligand dependent ERK activity waves have been reported in Drosophila tracheal development [72]. The progressive expansion of ERK activation in the tracheal placode is regulated by the outward spreading of EGFR ligand source mediated by the spreading of the expression domain of rho, the gene encoding an activator of the spi ligand. HeLa cells under apoptotic stress was also reported to induce the non-autonomous survival of neighboring cells by secreting FGF2 growth factor in a MEF-ERK dependent mechanism [40]. Our data further provided support for the idea that the transient ERK activation during apoptosis-induced survival of LECs is at least partly a ligand-dependent process. Interestingly, …….”**

**Furthermore, we have added statements about the limitations on our current work with ERK ligands, and also the interesting point that there are possibly two mechanisms that Vein acts to prevent cell death as the reviewer suggested. We added the following statement about a possible permissive function of Vein in the Discussion (lines 577-586). “However, our current work has only shown the effects of global downregulation of *vn* to reduce the ERK fluctuations in LECs, and has yet to demonstrate the dynamics of the Vn protein within the local LEC clusters during the transient upregulation of ERK in LECs. Another potential mechanism is that Vn and to a minor degree Spi, have a global permissive function where the presence of the ligand is prerequisite for preventing clustered cell elimination in the early phase independent of the regulation of the transient ERK activation. In this case, either: (1) the reduced ligand availability, or (2) the loss of EGFR availability and ligand-dependent activation due to the loss of endocytic activity can cause the global downregulation of the ERK signal to the level that cannot be overcome anymore by pulsed upregulation of the ERK for cell survival.”**

11/ Other studies have characterized ERK pulses near dying cells in the fly gut (Lucy O'Brien lab, Nature 2017, 10.1038/nature23678P), the human colon organoid (Pond et al. elife 2022, Paek lab, 10.7554/eLife.78837), or the secretion of FGF by dying HeLa cells (Bock et al. Nat Comm 2021, Tait lab, 10.7554/eLife.78837). I guess it would be fair to also quote them in this article..

**Our response
We appreciate the suggestion to include the references in our article. We have added them to our citations and list of references.**


Rev. 2:
Previously, the authors have shown that during abdominal morphogenesis, there are two phases of larval epithelial cell (LEC) elimination; an early phase, which is characterized by a slow elimination rate, and a late phase, which is characterized by a fast elimination rate (Hoshika et al., 2020). They also showed that this acceleration of cell elimination is due to a reduction in endocytic activity in the LECs, which regulates cellular myosin II organization, junctional E-cadherin levels, and caspase activation (Hoshika et al., 2020).

In this manuscript, Yuswan et al. now show that the acceleration of LEC removal is achieved by a change in apoptotic behavior of the LECs. They show that in the early phase, EGFR signaling is transiently upregulated in the neighbors of apoptotic cells, thus, limiting apoptosis to single-cell events. They also show that EGFR activity in the neighboring cells is EGF-ligand dependent. Lower endocytic activity and lower EGFR activity then promotes the switch to the late stage, in which LECs undergo clustered apoptosis where apoptosis is not limited anymore to single-cell events.

This manuscript establishes the role of EGFR signaling in the early and late stage well, however, the role of endocytosis in the process is less clear. The discovery of the switch between the two apoptotic modes is interesting. Some of the manuscript's results do not sufficiently support the conclusions, are not sufficiently interpreted, or are not sufficiently explained, as explained below:

**Our response**
**We thank Reviewer 2 for careful reading and being critical. Please find below our response to each specific comment.**

Major comments

1. The paragraph 'EGFR signaling activity decreases over time in LECs in an endocytosis-dependent manner' needs improving:

- Line 166: '…low level, as shown by the nuclear localization of miniCic, which further increased over time' - This statement might not be sufficiently supported by the data. A statistical test needs to be done to show that indeed, in Fig. 2F, the observed increase in nuclear localization in the controls are indeed statistically significant.

**Our response**

**We thank the reviewer for the constructive comment. We have performed statistical analyses for the comparison between different time points in control experiments and found that the nuclear/miniCic intensity indeed increases over time significantly. We have added the appropriate statistical significance between time points for control and the other genotypes in Fig. 2F.**

- Line 167: 'This observation shows that EGFR decreases globally throughout LEC elimination consistent with the decrease in endocytic activity' - This manuscript does not show how endocytosis changes over time. Thus, Hoshika et al., 2020 should be cited here. Even better, an analysis of an endocytic marker from 20 to 35 hours APF could be presented (Hoshika et al., 2020 do not present data for the whole time period discussed here).

**Our response**

**We thank the reviewer for careful reading. We agree that our current manuscript does not show the time evolution of endocytic activity. As the reviewer suggested, we have added further details (Lines 174-178) and cited Hoshika et al., 2020 to clarify the relationship between the downregulation of EGFR/ERK and endocytic activity.**

**“This observation shows that there is a global ERK activity decrease throughout the LEC elimination period. The dynamics of the decrease in ERK activity parallels with the decrease in endocytic activity in LECs, which was reported in our previous work [14]. While the reduction of endocytic activity ceases around 26 hAPF during the transition into the late phase [14], we observed that ERK activity continuously decreases even during the late phase.”**

**We also have tried to include the change in the Clc::GFP marker for the time at 35 hours but the analysis was technically challenging due to highly dynamic cell shape change as well as strong Clc::GFP signals for cell debris which scatter around all over the tissue at this very late stage (Response Fig. 3). Since we analyzed the time evolution of nuclear/miniCic intensity at 20, 25, and 30 hours APF, we think our data are sufficiently compatible with the data provided in Hoshika et al. in which the Clc::GFP was analyzed at time points between 20 to 29 hours.**

**Response Fig. 3. Confocal images of LECs expressing clathrin reporter CLC::GFP. Small punctae dot throughout LECs which gradually decrease to 26 hAPF. Starting from 29 hAPF, irregular aggregations of CLC signals from apoptotic cell debris (white arrows) starts to accumulate and obscure the LECs. Scale bar: 20 μm**

- Moreover, it could be made clearer that at this point in the manuscript, there is merely a correlation between the EGFR activity and endocytosis activity.

**Our response**

**Although we tried to accommodate the statement of a correlation between the EGFR activity and endocytosis activity in line 174, we found that it is rather redundant with the statement of the present sentence since we already stated, “This observation shows that EGFR decreases globally throughout LEC elimination consistent with the decrease in endocytic activity” (Line 174). Moreover, as we mentioned in our response to the next comment, we think that the statement about “correlation” between endocytic activity and EGFR/ERK activity would be an overstatement. We therefore prefer to keep its original shape, with some modifications as provided in the previous response.**

- Line 170: 'Abolishing endocytic activity by shiTS decreased average EGFR activity, especially in the early phase (Fig. 2D-F). - This statement needs to be improved, as only the early phase shows a significant difference compared to the control. Thus, the shits data does not sufficiently support the idea that EGFR activity and endocytosis activity correlate over time.

**Our response**

**We considered that the endocytic activity in the late phase is already low in control. Therefore, in the late phase, *shi* ^TS^ expression would not affect endocytic activity and consequently the EGFR/ERK activity as well. In contrast, it is expected that reducing endocytic activity in the early phase would affect the ERK activity. Indeed, we observed that the ERK activity is significantly decreased at 30 and 35 hAPF (early to mid) when *shi* ^TS^ expressed (Fig. 2D, F). As the reviewer suggested we also think that ERK activity and endocytic activity correlates. However, we did not want to mention a correlation between endocytic activity and ERK activity since we have not provided any data on directly showing endocytic activity. For this reason, we in fact refrained from stating “correlation” between endocytic activity and ERK activity in the original text.**

**Furthermore, rather than direct correlation between endocytic and ERK activities, the way we interpret the data is that endocytosis which helps ERK activation mainly in earlier phases. As the endocytic activity reduces, it does not help ERK activation in later stages We have modified the statement we presented in the Result part to more clearly reflect these interpretations as below.**

**Lines 180-181 “Abolishing endocytic activity by *shi*^TS^ decreased average ERK activity, which is limited in the early phase (Fig. 2D, F).”**

- Also, how I understand the presented data, the data can be interpreted so that shits leads to an early onset of LEC elimination, but that elimination itself is rather normal.

**Our response**

**We think that *shi* ^TS^ leads to an early onset of clustered cell elimination by skipping the phase where LECs undergo isolated single cell eliminations but not cell elimination in general. To confirm this notion, we have imaged *shi* ^TS^ from an earlier time point, 18h APF, before the known start of LEC elimination period. We found that indeed the LECs in *shi* ^TS^ undergo clustered cell elimination from the very beginning of the LEC elimination process (Response Fig. 4, Fig. S4B). This result shows that *shi* ^TS^ animals skip the isolated single cell elimination phase and it is not a mere early onset of LEC elimination process. We have included a statement describing this idea in line 376-378 as follows. “In contrast, inhibiting ERK signaling or endocytic activity increased the frequency of clustered LEC elimination even when the early phase initiated, skipping the phase with single cell eliminations (Fig. 6C-E, S4B-C).”**

**Response Fig. 4. Confocal imaging of shiTS expressing LECs**

**Imaging was obtained from 28 hAPF, 2 hours before the start of LEC elimination at 30 hAPF from the developmental delay caused by 18🡪29℃ incubation. White arrows indicate clusters of cells which are showing signs of apoptosis at 29 hAPF, and completed the nuclear breakdown at around 30h APF. Scale bar: 100 μm.**

- Line 171: 'Consistently, we also observed an increase in average ERK activity when endocytic activity was promoted by constitutively activating the endosome internalization regulating GTPase Rab5 (Rab5 Q88L) [36,37] (Fig. 2C, F)'. - This is imprecise, as there is no increase but the lack of a reduction in later stages. This suggests that there is a delay in elimination as stated by the authors in line 174: 'Rab5 Q88L significantly delayed LEC elimination compared to control for the time it took to reach half of the initial number of LECs(Fig. 2G-H).'.

**Our response**

**We thank the reviewer for the clarification. We originally intended to say that the EGFR/ERK activity which was low at the late phase became high by the expression of Rab5 Q88L. However, as the reviewer suggested it would be more intuitive to state that the high-level EGFR/ERK activity was maintained. We have rephrased the statement in line 181-184: “Consistently, we also found that over-activation of endocytic activity by expressing a constitutively active form of the endosome internalization regulator Rab5 GTPase (Rab5 Q88L) resulted in the maintenance of high average EGFR/ERK activity compared to control in the late phase [36,37] (Fig. 2C, E, F).”**

- The sample size in Fig. 2h is too small for reliable statistics. At least 5-6 data points each are needed.

**Our response**

**We thank the reviewer for pointing out the important issue. We have reanalyzed the timing of LEC apoptosis by plotting the time that a LEC disappears for all LECs from multiple pupae (control: 3 pupae, Rab5 Q88L: 5 pupae) throughout the elimination period. As presented in the figure below (Response Fig. 5), the average timing of apoptosis was significantly delayed in Rab5 Q88L expressing LECs in comparison to control LECs. We have replaced the half-life plot for Fig. 2H, 7D, and Fig. S5 additionally with these plots.**

***

***

**Response Figure 5. Survival time of LEC in control, Rab5 overexpressing (Fig. 2H), vnRNAi and EGFR RNAi (Fig. 7D), and spitz RNAi (Fig. S5) pupae. Each dot represents one cell. N = 3-5 pupae for each genotype group. Mann-Whitney test. ***P<0.001**

- Line 176: 'These results indicate that EGFR activity globally decreases over time and that the decrease of EGFR is regulated by the reduction of endocytic activity during LEC elimination.' - The data does not sufficiently support the conclusion that the decrease of EGFR activity is correlating with endocytic activity. The data can be interpreted in a way that EGFR activity and endocytic activity determine the onset of cell elimination.

**Our response**

**We thank the reviewer for the thoughtful comment. However, we are afraid that our data do not sufficiently support the idea that EGFR activity and endocytic activity determine the onset of cell elimination at the time of initial submission. We also do not conclude that the decrease of EGFR activity correlates with endocytic activity since we do not compare the levels of endocytic activity and the EGFR/ERK activity directly. Here, we just describe the experimental results that increasing endocytic activity by expressing Rab5 Q88L caused an increase of EGFR/ERK activity all over the tissue (Fig. 2F). In such a condition, LECs survive longer. By looking at the temporal pattern of cell elimination rate, one may discuss that the onset of rapid cell elimination phase delayed in Rab5 Q88L experiments as the reviewer suggested. However, we would refrain from mentioning it since we do not define the rapid cell elimination phase solely based on the cell elimination frequency. Instead, we show a correlation between EGFR/ERK activity with frequency of clustered cell elimination later (Fig. 6H). Moreover, as mentioned in our response above, we found that the *shi* ^TS^ experiment resulted in the skip of isolated single cell elimination phase (Fig. S4B). Although we cannot rule out the possibility that Rab5 Q88L expression delayed onset of rapid cell elimination, the reduced endocytic activity in control would regulate the timing of clustered cell elimination but maybe not the cell elimination in general.**

- It would be important to include the results of the important controls shown in Fig. S1 in the text. This figure is not mentioned in the text.

**Our response**

**We thank the reviewer for pointing out the lack of mention of Fig. S1. We have added the references to this figure at the appropriate sections.**

2. The data of miniCic fluctuations in Fig. 3 are not sufficiently explained for the reader to interpret und to understand them. The methods section does not explain how the fluctuations were analyzed.
- How was fluorescence intensity normalized?
- What does Fig. 3A show?
- How was the degree of fluctuation calculated? Which time period was considered to calculate the data shown in Fig. 3B?

**Our response**

**We apologize for the lack of explanation about our ERK fluctuation analysis. First, the fluorescence intensities of nuclear miniCic over time for each cell is normalized to its overall mean intensity. In Fig. 3A, we generated heatmaps based on this data, showing the evolution of ERK activity over time, which reveal the fluctuations. We interpret that the rapid changes from red (high miniCic, low EGFR) to blue regions (low miniCic, high EGFR) to be periods of transient ERK pulses. To further analyze the degree of ERK upregulation in Fig. 3B, we calculated the absolute change of miniCic between only the peaks and their next valleys in the wave, shown in the illustration below (Response Fig. 6) (absolute miniCic change between the magenta marked periods). For clarification, we have further added explanations in the relevant Results:**

**(Lines 214-218) “In order to obtain temporal profiles of the miniCic intensity for individual LECs, we automated the signal intensity measurements for the miniCic signal by combining with the tracking of nuclei labeled with a nuclear localized CFP::Venus protein. We found that individual LECs undergo multiple EGFR/ERK upregulation and downregulation events in pulses throughout the LEC elimination period (Fig. 3A left).”**

**(Lines 221-228) “To investigate whether these additional ERK dynamics are also regulated by endocytic activity, we analyzed the temporal evolution of miniCic nuclear intensity in the background of *shi*^TS^ and found that ERK activity fluctuations were diminished in *shi*^TS^-expressing LECs as well as *EGFR* ^RNAi^ (Fig. 3A right). We further analyzed the intensity of these transient ERK upregulations by measuring the average decrease of miniCic signal intensities in each ERK pulse (Fig. 3B top, see Materials and Methods for details). As expected, the degrees of ERK upregulation events were significantly diminished in *EGFR* ^RNAi^ and *shi* ^TS^ -expressing LECs”**

**Response Figure 6. Scheme of the analyses for the degree of ERK upregulation (Related to Fig. 3B). Only the change in miniCic intensities between the start and end (red dots) during the magenta regions are measured for the analysis. We define these drops in miniCic intensity as the ERK pulses.**

3. I am confused by the interpretation of the data shown in Fig. 3G. In line 216, the authors state that 'Interestingly, the average miniCic intensity increases up to 30h APF during LEC elimination (Fig. 3G), suggesting that the global EGFR/ERK activity decrease is independent of pro-apoptotic gene activity.', however, rhg-RNAi appears to affect miniCic nuclear localisation, suggesting that pro-apoptotic gene activity affects EGFR activity.
Also, the average miniCic intensity does not increase, as stated by the authors; it stays constant at a relatively high level without decreasing over time.

**Our response**

**We appreciate the critical comment about this point. We claim here that it is the basal and global EGFR/ERK activity that is independent of pro-apoptotic gene activity but we are not stating that the EGFR/ERK activity is unaffected by the proapoptotic gene activity. We think that the fluctuation is greatly reduced but a decreasing trend for the mean EGFR/ERK activity is still there since by looking at the heatmap in Fig. 3F, there is a clear tendency that the EGFR/ERK activity in LECs gradually decreases over time (from blue to red). We have now performed statistical tests for comparing mean values at different time points. The results have also shown that the decrease from 25 to 30 hAPF (but not 20 to 25 hAPF) is statistically significant. These results show that there is still a trend that the EGFR/ERK activity decreases over time even in the absence of pro-apoptotic gene function. However, we admit that the decrease of the average nuclear miniCic intensity became milder compared to control. To describe a milder decreasing trend in the RHG knock-down experiments, we have modified the relevant statement in line 238-243 as follows. “Interestingly, the average miniCic intensity started to increase at around 30h APF when LECs further migrate towards the midline (Fig. 3H), suggesting that the global ERK activity decrease is independent of pro-apoptotic gene activity. While these data suggest that the global basal ERK activity is regulated by a yet-unknown mechanism, we cannot still rule out a possibility that a low-level caspase activity downregulates the global ERK activity.”**

4. I do not understand the sentence in line 252: 'From these observations, we reasoned that fluctuations of EGFR/ERK may originate from the transient increase of EGFR/ERK in surrounding LECs.' - Do the authors mean that the observed fluctuations are mainly due to the fact that cells, that are neighbours of early cell death events, fluctuate?

**Our response**

**We thank the reviewer for asking the question. As the reviewer thinks, we intended to state that the observed fluctuation of the EGFR/ERK activity is derived not only from cells undergoing apoptosis but also the neighbor cells to these apoptotic cells. We think the poor readability of our writing may come from the lack of definition for the usage of “fluctuation” in our study. We defined that the “fluctuations” are the dynamic upregulation and downregulation of miniCic intensity which can be observed from the heatmaps we provided. We reasoned that the main contributors of these phenomena are the transient EGFR/ERK up- and down-regulations (pulses) in the surviving neighbours of apoptotic LECs. We have modified the sentence to improve clarity in line 293-296 as follows: “From these observations, we reasoned that the dynamically fluctuating ERK activities, which are represented in the heatmaps (Fig. 3A), mainly originate from the transient increase of ERK to induce the prolonged survival of LECs neighboring primary apoptotic LECs.”**

5. This sentence is unclear: Line 257: 'Together, these results suggest that the fluctuations of EGFR/ERK activity is the reflection of its transient increase in cell clusters….'. Do the authors again mean that the observed fluctuations are mainly due to the fact that cells, that are neighbours of early cell death events, fluctuate?

**Our response**

**Here, we also meant that the observed fluctuations are caused by the apoptosis-induced ERK pulses. We have further modified the summary sentence for clarification (line 302-304) as follows: “Together, these results suggest that the dynamic fluctuations in ERK activity in LECs are the results of transient increase in cell clusters. The transient increase in cell clusters may potentially prevent excessive apoptosis among adjacent cells in a short period.”**

6. A more detailed discussion of the results of Fig. 4F-l,J and how the authors interpret them is needed.

**Our response**

**We apologize for the confusion caused by our lack of detail in the discussion for the results mentioned. We have added further interpretations and explanations about our findings (also in addition to the response to #4-5 above) in multiple lines in the Results section (Lines 283-304)**

**“During the early phase, concurrent with LEC apoptosis, we observed a transient increase of ERK, represented by the transient decrease of miniCic, in several LECs neighboring the primary apoptotic LEC preceding apoptosis by about 60 minutes (Fig. 4A, F, J, Supplementary Movie 1).”**

**“Similar to the late phase in control pupae, we observed that even during the early phase, several cells in LECs exhibit concurrent decrease in ERK activity before their clustered apoptosis.”**

**“Careful observations revealed that in this low EGFR activity, small clusters of 3 or more cells undergo concurrent apoptotic events, which we determined by the near-simultaneous nucleus breakdown of neighboring cells. There are a few exceptional cases where multiple instances of apoptosis occur in a cluster within a little prolonged period (around 30 minutes) of the primary apoptosis (Fig. 4B’, G yellow line).”**

7. In the paragraph 'LECs neighboring a primary apoptotic LEC exhibit a transient increase in EGFR signaling activity', which begins in line 236, the authors show in Fig.4E that more cells die in the late phase, but this is not discussed in the text. It is only made clear that the behavior of the miniCic is changing in the neighbors of a dying cell, but not that the amount of dying cells changes. This is only explained later in the text. This is confusing.

**Our response**

**We apologize for the lack of explanation for the late phase observation in Fig. 4. We have added more in-depth explanation about this point as a part of the response to Reviewer’s comment #6 above. Specifically, we have added a figure showing this apoptotic pattern in Fig. 4B’. Furthermore, we have added the sentences detailing the increased cell deaths in a short period of time and the observation of concurrent apoptotic events during this late phase as provided in lines 289-293 for the response to #6.**

“**Careful observations revealed that in this low EGFR activity, small clusters of 3 or more cells undergo concurrent apoptotic events, which we determined by the near-simultaneous nucleus breakdown of neighboring cells. There are a few exceptional cases where multiple instances of apoptosis occur in a cluster within a little prolonged period (around 30 minutes) of the primary apoptosis (Fig. 4B’, G yellow line).**”

8. In Figure 5, no n-numbers are given.

**Our response**

**We appreciate the reviewer’s attention to detail. We have added the n-numbers for both control and OptoDRONC experiments used in the figure appropriately.**

9. Why are the data for the controls at 18ºC and 25ºC so different in Fig. 6L?

**Our response**

**We appreciate the reviewer’s concern about the differences in numbers of cluster elimination between 18ºC and 25ºC. We think that the frequency of cluster elimination may be affected by temperature to some extent. Along the same line, we suspected that the temperature shift to 29ºC for endocytic inhibition by *shi*^TS^ may also induce a minor increase on the cell elimination rate as seen in Fig. 1. Currently, we do not know the mechanism how the change in temperature affects the EGFR/ERK activity as well as the frequency of clustered cell deaths. However, we found that the different frequency of cluster cell deaths and the levels of EGFR/ERK activity in different experimental conditions would be useful to show the relationship between EGFR/ERK activity and clustered elimination. Therefore, we made use of this fact to correlate the EGFR/ERK activity with the frequency of clustered elimination.**

10. The observation of cluster cell death is very interesting. It would be helpful to see data with a membrane marker to understand the impact of cluster cell death on the integrity of the epithelium. As far as I understand, neighboring LECs do not delaminate at the same time (probably to avoid a rupture in the tissue).

**Our response**

**We are pleased that the reviewer finds this point interesting. Currently, we have only presented and defined clustered LEC deaths as the concurrent loss of 3 or more neighbouring nuclei without a full investigation in the integrity and mechanics of the membrane itself. We have performed imaging with a membrane marker as seen in some of the examples below (Response Fig. 7).**

**Response Fig. 7. Clustered apoptosis in LECs shown by membrane marker Nrg::GFP in either control (top two rows) or EGFR RNAi (bottom two rows) over time. Cyan labeled cells are those undergoing clustered apoptosis. White arrows indicate the sealed junctions following the complete extrusion of apoptotic LECs. 0 hour is when cell extrusion completes. Scale bars: 50 μm.**

**From the figures, we observed no epithelial rupture following cluster eliminations in control as well as in EGFR RNAi. Cell eliminations in a cluster occur in a concurrent manner but not in sequence. The epithelial sealing seems to be concurrent with the apical constriction of the apoptotic cells. We noticed that LECs are highly motile and dynamically changing shapes in the late phase in control pupae. We think that the tissue integrity is maintained despite of the concurrent loss of cells due to the flexibility of surrounding cells and tissue fluidity. It is interesting but is not the focus of this work. We would like to refrain from disclosing this observation in this manuscript and publish anywhere else since we would need more thorough analysis to address the mechanics of epithelial sealing.**

11. The cluster analysis is crucial for this manuscript. The way the clusters were identified means that, in a cluster of four cell deaths, two of the cell deaths might be two time points apart (and thus not clustered?). I wonder whether the way the analysis was done does merely reflect that, in the late stage, many more cells die and thus statistically more cells die in the vicinity of each other. Could the authors explain better how the analysis was done and how this provides evidence for clustering?

**Our response**

**We thank the reviewer for the constructive comment. Our current analysis defines that neighboring cells which lost nuclear signals at the same frame or at the next frame (10-minute interval), they are included in the cluster. Using the example the reviewer**

2

**provided, if the two adjacent pairs of cell death occur at the 15^th^ and 16^th^ frames, then they are considered in the same cluster. However, if they happen at the 15^th^ and 17^th^ frames (20-minute difference), they are separate pairs of apoptosis, although still notable as the example can be seen in late control in Fig. 4B. The one frame detection lag is introduced to avoid the effect by a potential artifact of a nucleus detection algorithm that we used. More precisely, we found some cases in which a nucleus mistakenly remained detected after nuclear breakdown which is obvious to human eyes. This is usually due to the remaining signal still being relatively high compared to the background, as presented in the example figure below (Response Fig. 8).**

Tracking remains despite apoptosis

t_1_

t_0_

t_-1_

t_-2_

**Frame 0 (apoptosis)**

**Frame +1**

**Frame -1**

**Frame -2**

*****

*****

*****

*****

*****

**Response Fig. 8. Example of Trackmate maintains LEC nuclear tracking in the next frame after apoptosis. Magenta circles track the LEC nuclei, the last frame when LEC nuclei are marked is defined to be an apoptotic event (here apoptotic cells are marked with asterisks). White arrows indicate the LEC which was still being tracked at the next frame after apoptosis, despite clear nuclear breakdown.**

**Therefore, to ensure the detection of nuclear loss being closer to the one by human eyes, we considered the cases neighboring cells which lost nuclear signals at the same frame as well as at the next frame as concurrent cell death.**

**In order to test whether the cluster cell death is a mere consequence of increased density of dying cells, we have run simulations which iterate and randomly eliminate cells with the elimination rates that we observed in experiments in control, EGFR RNAi, and *shi* ^TS^ as explained in our response to Reviewer 1 for the Major comments #1-3.**

Also, some more information about the clusters would be helpful: Are there multipe clusters at the same time? Do clusters have a certain shape? How many cells form a cluster?
Moreover, the authors do not state the time interval in the movies used for the cluster analysis in Fig. 6. This is important to assess to what extent cell death in two consecutive frames can be considered to be dying at the same time.

**Our response**

**We thank the reviewer for pointing out uncertain description of the cluster cell death. The time interval of our movies is 10 minutes. This leads us to consider that clustered apoptosis can be determined by the breakdown of nuclei in neighboring cells which occur at the same frame and within 1 frame (10 minutes) from the selected primary apoptosis. We also determined that the apoptosis of 3 or more cells are considered clustered, which can be in a line of cells or adjacent to each other (see Supplementary Fig. 4A bottom right). For example, if there are 4 nuclei in line, with two nuclei breaking down at t0, and the other 2 adjacent nuclei at the next frame, we consider these LEC apoptosis as clustered with the reasons we described in Response Fig. 8. These conditions became the base for our analysis for the clustered apoptosis. We have detailed our description in line 669-677.**

Other comments
Line 139 'indicate that EGFR expression has an inhibitory effect against LEC elimination' should be 'indicate the EGFR activity has…', I think.

**Our response**

**As the reviewer pointed out, the inhibitory effect was the result of EGFR activation since we used a constitutively active form of EGFR in this experiment. We have changed the text accordingly.**

Fig. 1 - Graph in top right lacks a y-axis label. In E, wrong color for EGFRtop graph.

**Our response**

**We apologize for the mistakes in labeling and plotting. We have added the appropriate labels and coloring to these graphs.**

Line 166 '…low level, as shown by the nuclear localization of miniCic, which further increased over time' - It would be clearer if the authors would use two sentences to make this point clearer: '…low level, as shown by the nuclear localization of miniCic (Fig. 2B, F)). This nuclear localization further increased over time (Fig. 2B, F)'.

**Our response**

**We agree with the comment. We have rephrased the sentence accordingly.**

Fig. 2F - The y-axis label could be clearer, e.g. 'Ratio nuclear/cytoplasmic miniCic intensity'.

**Our response**

**We have clarified the y-axis labels as suggested by the reviewer.**

Line 199: '…fluctuates differently for each cell (Fig. 3A top, B top).' - should only be 'B'.

**Our response**

**We appreciate the reviewer’s attention to detail. It was a typo when intended to refer to the control as “B left”. We have removed “B top” in the legends.**

Is Fig. 3D showing nls::CFP::Venus (stated in the figure) or nls::SCAT3 as stated in the legend?

**Our response**

**We apologize for the confusion. While the nls::SCAT3 contains both CFP and Venus tag, it is more accurate to state it as nls::CFP::Venus, and we have modified the legend as such.**


Rev. 3:
In this manuscript, Yuswan and the team investigate the apoptosis mechanism of larval epidermal cells (LECs) during Drosophila metamorphosis. Expanding on their prior discovery of decreased endocytic activity in LECs leading to increased apoptosis, the authors show the crucial role of the epidermal growth factor receptor (EGFR) signaling pathway in governing both endocytic activity and LEC apoptosis. Additionally, they demonstrate that EGFR in neighboring cells prevents cluster apoptosis. Although this protection mechanisms via ERFR signaling were documented in other contexts, this study introduces a novel concept where developing tissue regulates apoptosis modes to meet specific developmental needs, such as the requirement for clustered apoptosis to facilitate the growth of neighboring tissue. Notably, this is distinct from the traditional view that cluster of apoptosis is disadvantage for maintaining tissue barrier function.
While the research question is intriguing, concerns arise about the clarity of explanations and supporting evidence for critical data, suggesting that the manuscript may be somewhat premature in its current stage.

**Our response**
**We thank Reviewer 3 for showing the interest to our manuscript and careful reading. Please find below our response to each specific comment.**

MAJOR COMMENTS:
#1. The use of two different methods to analyze EGFR activity introduces confusion. Analyzing some data as the ratio of nuclear to miniCic intensity (Figs. 2, 3, 6) while analyzing others as miniCic intensity (Figs. 4, 5) makes it challenging to intuitively link the two measures. For example, when miniCic intensity increases, the ratio indicating ERK activity could either rise or fall, depending on alterations in nuclear intensity. Conversely, the value of miniCic intensity, representing ERK activity, increases as the miniCic intensity of the image rises. To enhance clarity, I recommend maintaining consistency in the analyses, unless there are specific reasons for the variation, which should be explicitly clarified in the text. A side note: Fig. S1 uses nuclear to 'cytoplasmic' miniCic intensity as the third measure of EGFR activity.

**Our response**

**In order to measure the ratio of nuclear to cytoplasmic miniCic intensity for most experiments, we manually detected nuclear and cytoplasmic signal of miniCic for individual cells. We performed the measurement manually since we needed careful and accurate measurement for experiments such as local clusters of cells and we were not able to implement an automated detection of the cytoplasmic intensity for a given nucleus in a reliable way. On the other hand, for the analysis of fluctuation of the EGFR/ERK, it is required to track the miniCic signal for all cells for long time. To this end, we opted a simple automated signal detection by an imageJ plugin, TrackMate. We tracked all nuclei and measured miniCic in all of the tracked nuclei by the TrackMate. We noticed that the change in cytoplasmic signal intensity is only marginal. Therefore, we used the nuclear miniCic as a proxy for obtaining an outline of the EGFR/ERK activity to capture the fluctuating feature for miniCic signals (Response Fig. 9). We have added the appropriate explanation for the differences in analyzing EGFR/ERK activity in the relevant sections (Fig. 3)(lines 214-218)**

**“In order to obtain temporal profiles of the miniCic intensity for individual LECs, we automated the signal intensity measurements for the miniCic signal by combining with the tracking of nuclei labeled with a nuclear localized CFP::Venus protein. We found that individual LECs undergo multiple EGFR/ERK upregulation and downregulation events in pulses throughout the LEC elimination period (Fig. 3A left).”**

**Response Figure 9. Nuclear miniCic intensity obtained from TrackMate data normalized to the average for each cell.**

#2. In Fig. 2B, it is hard to see the distribution of miniCic as shown in Fig. 2A. To enhance visibility, it would be beneficial to include a composite image of the green (nls::CFP::Venus) and purple (miniCic::mScarlet) channels, at least for the wild-type. This additional representation would provide a clearer visual understanding of the miniCic localization, especially in the cytoplasm.

**Our response**

**We thank the reviewer for the suggestion. To provide a clearer visual information of the miniCic localization, we have added a composite image for the control incubated at 25^o^C in Fig. 2B labelled as Merge.**

#3. It is challenging to remember high nuclear/miniCic intensity represent low EGFR activity and vice versa. It would be helpful to include an indicator, similar to the one in Fig. 4E, in Fig. 1F.

**Our response**

**We want to clarify if the reviewer intended to mean Fig. 2A for the EGFR/ERK high/low indicators, instead of Fig. 1F. Assuming so, we have added an additional indicator scheme in Fig. 2A to explicitly show the miniCic signal intensity relationship with ERK activity.**

#4. Fig. 3A. It raises questions about the observation of more blue (indicating high EGFR activities) during 30h and 35h APF. This is opposite from the trend shown in Fig. 2F. Please clarify.

**Our response**

**The issue that more blue area is noticeable even after 30h was derived from the effect of data visualization. White color (midpoint value) was assigned to the average miniCic intensity of all cells, rather than the one of each cell. Because of this way of color assignment, the midpoint value (white color) was higher than 1.0. We have scaled the colour indicators appropriately and set the midpoint to 1.0. We have also labelled the extremes as “Low” or “High” nuclear/cytoplasmic miniCic signal ratio or simply nuclear miniCic signals. As can be seen in the appropriately scaled heatmaps, the amount of blue is considerably less after 30h APF as the EGFR activity overall becomes lower during the late phase.**

#5. Fig. S2D. Including a plot of miniCic intensity for surviving cells would be beneficial. This additional data would provide insights into the timing of EGFR activities in surviving cells relative to caspase activation in dead cells.

**Our response**

**We appreciate the suggestion to include the miniCic data for the surviving cells. This data have been added to the plot (Response Fig. 10). It seems that the transient ERK upregulation occurs later than the initiation of apoptotic caspase activity raise. We also have provided a few discussion points in the Results section to describe our interpretation of the temporal relationship (lines 286-287) as follows: “This ERK pulse initiates during the gradual decrease of ERK and around the start of the apoptotic caspase activation (Fig. S2D).”**

6

5

4

3

2

1

2.0

1.8

1.6

1.4

1.2

1.0

0.8

-2:00

-1:30

-1:00

-0:30

0:00

Normalized GC3Ai intensity

Normalized miniCic intensity

Time to apoptosis

0

**Response Fig. 10. Updated Fig. S2D plot which includes GCAi and miniCic from the apoptotic cell, and miniCic from the surviving neighbor cells.**

#6. In Fig. 4A, EGFR/ERK upregulation is observed in several neighboring larval epidermal cells (LECs); however, not all neighboring cells exhibit this upregulation. It would be insightful to discuss potential mechanisms for this differential response in the discussion section, addressing why some neighboring cells show upregulation while others do not.

**Our response**

**As the reviewer pointed out, we did see some cells which do not seem to exhibit any EGFR upregulation upon neighbor apoptosis. We speculate that the junction / contact length may have an influence in modulating the degree of EGFR activation in neighboring cells, or that it may be a stochastic process. We have added a couple of sentences to add some discussion about this point in relation to the potential mechanisms we proposed in the Discussion section (lines 586-595).**

**“Lastly, while previous studies show that the ERK waves propagate multiple layers of cells [38], the ERK pulses in LECs are confined to only the cells adjacent to the primary apoptotic cells. In addition, all adjacent neighbor LECs do not necessarily undergo the ERK pulses. Interestingly, a recent report showed that mechanostransduction of YAP signaling only in the largest neighbor cells induce compensatory proliferation in cell culture [78], suggesting a similar mechanism may play a role during LEC elimination. Understanding the precise mechanism by which ligands regulate cluster LEC elimination awaits further analyses.”**

#7. It would be helpful to show that tissue integrity remains uncompromised following cluster apoptosis by including E-cad data fomr the wild-type late phase.

**Our response**

**We have found no signs of compromise of tissue integrity following clustered elimination of LECs, as can be seen in the Response Figure 6 to Reviewer 2 above.**

#8. It's not clear why most of the data starts from 20h APF, but Fig. 1I begins from 30h APF? To ensure consistency, it would be helpful to either explain the reasoning for this discrepancy or provide data from 20 hours APF for Fig. 1I.

**Our response**

**We apologize for the confusion about the difference in the initial time for imaging. This is due to the difference in incubation temperature for different experiments. *shi* ^TS^ animals were raised at a permissive temperature (18˚C) until early pupal stage and then shifted to a restrictive temperature (29˚C) in order to analyze the effect specifically on LECs at a time window where they undergo apoptosis. Since there is developmental delay in pupae incubated at a low temperature in general, LEC elimination starts at 30h APF at 18˚C unlike the ones incubated at 25˚C in which the elimination starts at 20h APF. We have clarified the reasoning of the late start of imaging in the Materials and Methods section (line 621-623), and also added statements in the relevant Results paragraphs (line 137-139). “To accommodate the developmental delay due to temperature shift from 18℃ to 29℃, we started our imaging from 30 hAPF (See Materials and Methods).”**

#9. While I understand that optoDRONC induces apoptosis in several cells but not single cell, the manuscript lacks information regarding the extent of the affected area through optogenetics. It would be valuable to estimate the size of the region where optoDRONC can induce apoptosis. Consider expressing optoDRONC in other tissues, such as histoblasts, and employing the same laser settings to monitor the number of cells undergoing apoptosis. This approach could provide insights into the spatial reach of optoDRONC in inducing apoptosis.

**Our response**

**We tried the exposure of histoblasts to a 488 nm laser. However, we were unable to see any apoptotic events in the histoblasts upon the laser exposure. For LECs, we found that the ROI we used (25x5 um, approximately the size of one nucleus of LECs) induced OptoDRONC in several LECs within 100 um range (Lines 330-332 “Due to technical limitations, we selected settings that allowed us to consistently promote OptoDRONC-induced apoptosis of several cells (approximately 100 μm away at maximum)”.**

#10. It's hard to see "transient EGFR/ERK activation (line 287)" in the figure. To enhance clarity, I recommend making this phenomenon more visually by incorporating arrows, ellipses, or other graphical elements.

**Our response**

**We apologize for the lack visual cues to explain our observations here. We have added arrows to show which survivor cells receive the transient EGFR/ERK activation (Fig. 5A-B).**

#11. The statement that "EGFR/ERK activity increases locally and transiently in the cells adjacent to an apoptotic cell (line 390)" is made, but to support this claim, I recommend checking whether the next-nearest cells do not exhibit an increase in EGFR/ERK activity.

**Our response**

**From our preliminary observation we saw that the 2^nd^ layer of neighbors do not exhibit the EGFR/ERK pulses however there is a need for a more in-depth investigation and we have added a few statements in the discussion as follows:**

**(Lines 586-588) “Lastly, while previous studies show that the ERK waves propagate multiple layers of cells [38], the ERK pulses in LECs are confined to only the cells adjacent to the primary apoptotic cells. In addition, all adjacent neigbor LECs do not necessarily undergo the ERK pulses.”**

#12. It is not clear which data shows "vn regulates the transient EGFR activation in cells adjacent to the apoptotic LEC (line 363)". The reduction in EGFR fluctuation with vn RNAi, as shown in Fig. 7E, is not specific to the cells next to the apoptotic cell. Additionally, the statement, "We further identified that the EGFR ligand vein (vn) is a key modulator of the transient EGFR/ERK upregulation that promotes the survival of cells neighboring apoptotic LECs (lines 398-400)," appears to be an overstatement. To ensure accuracy, it is recommended to rephrase the text.

**Our response**

**We thank the reviewer for pointing out the overstatements in our text. From the vn RNAi experiments, we interpreted that the loss of EGFR/ERK fluctuations is caused by the loss of apoptosis-induced ERK pulses on the background of vn RNAi similarly to the data from RHG knock-down. However, we admit that we have no evidence that the ERK pulses are caused by the loss of local interaction between neighboring LECs for vn RNAi. We have omitted the statement that attributes the transient EGFR/ERK activation to the interaction with adjacent apoptotic LECs in Results (line 439-442): “In summary, our results showed that *vn* regulates the transient ERK activation in LECs and prolongs their survival, suggesting that the transient upregulation of ERK in LECs, which prevents clustered cell deaths and prolongs their survival during the early phase, is partly a ligand dependent process.” and in Discussion (line 477-479): “We further identified that the EGFR ligand vein (*vn*) is a key modulator of the transient ERK upregulation that promotes the survival of cells, thereby showing that the transient ERK activations is at least partly a ligand dependent process.”. Moreover, as we wrote in our response to the minor point 10 by Reviewer 1, we discussed another possible mechanism which is a global permissive function of Vn in regulating clustered cell elimination (line 580-593) as follows. “Another potential mechanism is that Vn and to a minor degree Spi, have a global permissive function where the presence of the ligand is prerequisite for preventing clustered cell elimination in the early phase independent of the regulation of the transient ERK activation. In this case, either: (1) the reduced ligand availability, or (2) the loss of EGFR availability and ligand-dependent activation due to the loss of endocytic activity can cause the global downregulation of the ERK signal to the level that cannot be overcome anymore by pulsed upregulation of the ERK for cell survival…..”**

#13. In the discussion, particularly from line 407 to 432, there is a mixing of perspectives, shifting from a global standpoint (ecdysone) to a local one (caspase) and then returning to a global perspective (global change in junctional tension). This creates confusion for the reader, making it challenging to understand the connection between the discussion points and either global ERK decrease or local ERK fluctuation. To enhance clarity and coherence, I recommend rearranging the order of this section of the discussion, ensuring a logical flow that helps the reader better follow the linkages between the different perspectives on ERK regulation.

**Our response**

**We thank the reviewer for the constructive comment. We have rearranged the flow in a manner that allows the readers better follow the logic and the linkages between the different perspectives on ERK regulation. We first introduce potential regulation by ecdysone as a global point of view. Then, we still maintain the global view but from another perspective by discussing a mechanical regulation. Finally, we discuss a mechanism that could accelerate the reduction of endocytic activity through a positive feedback loop in individual cells. Now we are going from global to local standpoint. We believe this change in flow has increased clarity and coherence of our statements. Please refer to the main text of our revised manuscript in lines 515-552.**

MINOR COMMENTS:
#14. The labels on the x- and y-axes are either missing or unclear in the following figure.
- Fig. 1A right
- Fig. S2D. What's the unit?
- Fig. 6F-H. What's the unit?

**Our response**

**We have clarified the units and labeling for these figures. Additionally for clarification, we have modified and rearranged Fig. 6, with the old Fig. 6F-H now correspond to Fig. 6E-F**

#15. Some still images and graphs use hours as the unit, while others use minutes. Please standardize the unit throughout for consistency.

**Our response**

**We apologize for the inconsistencies. We have changed all of the units to use hours for all the plots.**

#16. Fig. 2C-E. Change the order so that it aligns with the sequence in which they appear in the text. In the text, Rab5^ Q88L appears later than shi^TS.

**Our response**

**We have rearranged the figures so it shows shi^TS before Rab5^Q88L.**

#17. It would be informative to include heatmaps depicting the nuclear miniCic::mscarlet intensity over time for rhg^RNAi, similar to the presentation in Fig. 3A.

**Our response**

**We appreciate the suggestion. We have added the heatmap for miniCic intensity in RHG RNAi LECs as Fig. 3F**

#18. The following sentence lacks statistical support.
- "the average miniCic intensity increases up to 30h APF… (line 216)"

**Our response**

**We have added the statistical significance between each time point in both 25C and 18-29C controls (20h vs 25h and 25h vs 30h) to the relevant Fig. 2F to support our statement.**

#19. "Previous reports have observed the upregulation of EGFR/ERK in clusters of cells in response to apoptotic events [30,38], and our data also indicate that the fluctuation of EGFR/ERK requires the initiation of LEC apoptotic events (line 238)." The mention of EGFR in both neighboring cells and apoptotic cells in a sentence can be confusing.

**Our response**

**We appreciate the helpful comment. We have split the sentences for clarity as suggested. (Lines 273-275)**

**“Previous reports observed the upregulation of ERK in clusters of cells in response to apoptotic events [30,38–41]. In support of this notion, our data suggest that the fluctuations of EGFR/ERK activity are suppressed by the inhibition of LEC apoptosis (Fig. 3C-H).”**

#20. "We observed a transient increase of EGFR/ERK in … (line 248)." Adding the clarification about the transient decrease of miniCic intensity, such as 'a transient increase of EGFR/ERK, accompanied by a transient decrease in miniCic intensity, in …,' enhances the reader's understanding.

**Our response**

**We appreciate the suggestion. We have added a phrase to clarify that we interpreted the transient decrease of miniCic in surviving LECs to be the transient increase of EGFR/ERK activity as such:**

**Line 283-285 “….** **we observed a transient increase of ERK, represented by the transient decrease of miniCic, in several LECs neighboring the primary apoptotic LEC preceding apoptosis by about 60 minutes”**

#21. It appears that Fig. 4A (and 4B) may not correspond to Movie 1 (and 2). I recommend using the same data and magnification for both the still images in the figure and the movies to ensure consistency.

**Our response**

**We have matched the movies and the still images for this figure.**

#22. It would be helpful to indicate the primary apoptotic cell in Movie 1 and 2.

**Our response**

**We have added an arrow which indicate the primary apoptotic cell which we used in our analysis.**

#23. I would suggest revising the following wordings.
- "within roughly 60 minutes of primary LEC apoptosis (line 249)". The word 'within' is confusing. It would be more clear to use 'proceeding'.
- "EGFR upregulation in the LECs that initiated apoptosis (line 289)". It would be clearer to state 'EGFR upregulation in the LECs associated with induced apoptosis'.
- "we reasoned that fluctuations of EGFR/ERK may originate from the transient increase of EGFR/ERK in surrounding LECs (line 252)". Please specify which fluctuation are you refiring to about the first 'fluctuation' in this sentence. Is it fluctuation in the primary dying cell?
- "non-linearly changes the state of cells." It is not clear what 'non-linearly' means.

**Our response**

**We apologize for the confusion. We wanted to indicate that the ERK pulses occur within a range of time (starting from 60 mins before primary cell apoptosis). We replaced the wording with “preceding” as suggested and further added details for clarification of these points as provided in the Line 284-285.**

**We have replaced the line "EGFR upregulation in the LECs that initiated apoptosis” with Lines 337-338 “Interestingly, we observed that ERK upregulation in the LECs that underwent the induced apoptosis did not arrest the apoptosis process,**

**We have modified the line “we reasoned that fluctuations of EGFR/ERK may originate from the transient increase of EGFR/ERK in surrounding LECs (line 252)” with the lines 293-296 “From these observations, we reasoned that the dynamically fluctuating ERK activities, which are represented in the heatmaps (Fig. 3A), mainly originate from the transient increase of ERK to induce the prolonged survival of LECs neighboring primary apoptotic LECs.”**

**Basically, we wanted to mention that the cell state transition is accelerated by a positive feedback loop between endocytic activity and caspase activations in LECs. However, “non-linearly” was not properly introduced with sufficient explanation. We have removed the “non-linearly”. Instead, we have introduced a lengthy but more detailed description to convey a meaning that we wanted to introduce by saying “non-linearly” in lines (506-510) “In a similar mechanism, the endocytic activity in LECs may be downregulated by caspase activations. Combined with our results as well as our previous study [14], this interaction is considered to form a positive feedback loop, further decreasing endocytic activity. Consequently, this reduction would accelerate the transition of the cell state.”**

#24. n number is missing in Fig. 5.

**Our response**

**We have added the appropriate sample numbers in the legend for Fig.5.**

#25. I don't see the term 'cyan ellipses' mentioned in the legend of Fig. 5 corresponding to the images.

**Our response**

**We have removed the ‘cyan ellipses’ from the Fig. 5 legend, and replaced them with arrows which point at the survivor cells in Fig. 5 A-B are the surviving cells used in the miniCic intensity analysis, which we added an additional explanation in the Materials and Methods section (specifically, Manual analysis for nuclear miniCic intensity in cell clusters) as follows. Lines 665-666 “For the optogenetic analysis, we included all the surviving cells neighboring the OptoDRONC-induced apoptotic LECs for the miniCic analysis.”**

#26. What does 'the degree of transient EGFR activity... (Fig. 6I)' mean, and how does it relate to the content presented in Fig. 6I?

**Our response**

**We mistakenly labelled the figure as Fig. 6I, where it should have been Fig. 6H. It is now Fig. 6G since the figure panels have been remodeled. For the statement, we have appropriately added extra sentences “The lower the average ERK activity was, the more clustered eliminations were observed throughout all experiments irrespective of developmental time, supporting the idea that ERK activity protects cells from mass apoptosis in clusters.” (line 394-396) to clearly relate EGFR and clustered apoptosis.**

#27. I suggest overlapping the data of EGFR RNAi with Fig. 7F to maintain consistency with the other graphs in Fig. 7. The same comment applies to Fig. 6E.

**Our response**

**We appreciate the constructive comment. We have replaced the bar plots showing the number of cells undergoing clustered apoptosis in Fig. 6 and 7 with overlaid line plots for clarity and consistency with the units. Additionally, we have modified the explanations for these figures in the Figure legends.**

#28. Only scientists involved in histoblast expansion may comprehend 'the proliferating cells of neighboring tissue, which increase exponentially and thereby replace the shrinking tissue more rapidly as tissue remodeling progresses (line 444).' I recommend adding more detailed information, including 'Histoblast', to facilitate understanding for non-experts.

**Our response**

**We have specified the exponentially growing cells in our model to be histoblasts as provided in this change:**

**(Line 525-530) “Despite its limitations, our study constitutes the first example of implementing clustered cell elimination in the context of development and demonstrates that it plays an active role in accommodating the proliferating cells of neighboring tissues – in our model, the Drosophila adult histoblasts – which increase exponentially…..”**

#29. I don't see a "sharp transition (line 449)" in the phenomenon observed.

**Our response**

**We considered it as a ‘sharp transition’ since the transition between two phases (early and late phases) in our definition does not take hours. However, we admit that it may not necessarily be a sharp transition for readers. To avoid any confusion that might be caused by this expression, we have changed it to just a ‘transition’.**

#30. Typo
- Reference (lines 92 and 100).
- Fig. 2G (line 137) should be Fig. 1G.
- Fig. 2H (line 138) should be Fig. 1H.
- Fig. 3D-E (line 211) should be Fig. 3C-E.
- Fig. 6C (line 282) should be Fig. 5C.

**Our response**

**We appreciate the reviewer for finding the mismatches. We have fixed and appropriately referred the figures in the texts.**

#31. The final sentence of the abstract, "These findings reveal the tight temporal regulation of tissue remodeling, accomplished by mode switching via local cell-cell communication." is too general and doesn't adequately signify the novelty of the study. I suggest revising this sentence to better capture the unique contributions of the research. Please see my summary paragraph above for more detail.

**Our response**

**We appreciate the critical suggestion to improve the main message of the research in the abstract. We have modified our end statements in the abstract (lines 51-53), and added a few discussion points (Lines 530-532):**

**“The role of clustered elimination in this context is distinct from the conventional understanding, that clustered apoptosis is disadvantageous by compromising tissue integrity. Our study on the molecular mechanisms regulating LEC clustered apoptosis…”**

**We hope this reflects the novel idea that the regulation of clustered elimination is advantageous in special tissue remodeling contexts, which differ from the traditional view of the disadvantageous clustered elimination.**
