# Supplementary material for: Reduction of endocytosis and EGFR signaling is associated with the switch from isolated to clustered apoptosis during epithelial tissue remodeling in Drosophila
Source: PLoS Biol. 2024 Oct 14;22(10):e3002823. doi: 10.1371/journal.pbio.3002823 (PMC11472926; doi:10.1371/journal.pbio.3002823)
Supplement: Attachment — Submitted filename: EGFR_rebuttal2_submitted.docx [file pbio.3002823.s014.docx]

Responses to reviewers’ comments

We would like to thank all the reviewers for the constructive comments. We have further revised our manuscript based on their comments. We added our responses and highlighted the modified/added statements with red characters in the main text.

Rev. 1:
I appreciate the effort of the authors to answer my points and they have now included interesting complement in the current manuscript which is really improved. I should say though that as a matter of fact, my points 1 and 3 that I raised are not fully sorted by what is proposed by the authors. I am sorry to insist on those, but since most of the articles is about the clustering and ERK dynamics, I believe they need to be well set.

In point 1, I was arguing that it is difficult to prove that the clustering of cell death has a physiological function (and accelerates tissue remodeling) as claimed by the authors. To prove this, one would need to compare two conditions with exactly the same death rate, but with one randomly distributed, and one clusterised, and see how this affect the developmental process. Without this, all we can say is that there is a correlation between the appearance of clusters and an acceleration of LEC elimination, and this is not possible to state any causal link. So I remained convinced that it is very hard to prove that the "clustering" of cell elimination has a function per se. This does not remove the interest of most of the observations, but I still believe that the title of the manuscript is misleading as there is no proof that cell death clustering "accelerates" tissue remodeling (the best one can say is that clustered death "is associated with" accelerated tissue remodeling).

**Our response**

**We would like to thank the reviewer for the constructive discussion. We agree that it is difficult to state causal relationship between the cluster elimination and acceleration of the tissue remodeling. Our observations with the Rab5 overexpression showed that there is still a rapid LEC elimination phase despite being significantly delayed, despite no clustered cell deaths within the live imaging period. This result indicates that there may be other factors which can influence increase in the rate of LEC elimination. These factors may contribute to the acceleration of the tissue remodeling process together with the clustered cell deaths. We also admit that the clusterization *per se* would not accelerate the tissue remodeling. It is not just a clusterization but it is a local propagation of cell eliminations triggered by single primary apoptosis events that would increase the number of eliminated cell associated with each apoptosis event and therefore accelerate the tissue remodeling. If there is no such propagation of cell death, only single cell death would occur resulting in no acceleration of tissue remodeling. Nevertheless, as the reviewer pointed out we still cannot directly test the contribution of cluster cell death to tissue remodeling by experiment, it would be too strong to state cell death clustering "accelerates" tissue remodeling. We therefore revised the title to “Switching to clustered apoptosis induced by reduction of endocytosis and EGFR signaling is associated with the acceleration of tissue remodeling rate”**

In point 3, I was pointing at the difficulty to sort the contribution of global ERK modulation and ERK pulses and their link with clustered death. The authors provide now interesting simulations showing that clustered death is much more frequent than what would be expected from a random distribution, and this for any condition (even for the control at early stage). This is per se very interesting as it suggests that there is a natural tendency to have clustered apoptosis (maybe because of underlying pattern that kill cells in specific domains ?), and open another unresolved question of why is there higher clustering than expected from random. However, this does not really address my point which is to sort the relative contribution of basal ERK activity (and thus basal rate of cell death) versus ERK pulses for the regulation of cell death cluster in the late pupae, EGFR depleted and shiTS conditions. To really address this point, **the authors could maybe compare the relative
proportions of clusters in between control, EGFR RNAi and ShiTS in the simulations (which will estimate the fold increase of clusters number expected just based on changes in the rate of cell death)**, and then do the same for experimental data from Figure 6E (looking at fold increase of clusters relative to the WT). Assuming that ERK fluctuations plays some role in cluster occurrence, then one would expect higher fold change of cluster numbers in EGFR RNAi and ShiTS relative to experimental control compared to the fold change obtained in the simulations (in summary, comparing "relative" frequency compared to control and not absolute numbers, which hide difference because of this natural tendency of clustered death). Right now, the data in Figure 6G would tend to suggest that most of the clustering behaviour could be explained by ERK basal activity (and thus is not really giving strong argument for the contribution of ERK fluctuations/pulses in the regulation of death
clustering).

**Our response**

N.S.

N.S.

N.S.

*EGFR ^RNAi^*

Control 25°C

*shi ^TS^*

Ratio of clustered apoptosis to all apoptosis (a.u.)

**Response Fig. 1. Ratio of clustered apoptosis numbers to all apoptosis in each phase. Student’s T-test, NS not significant**

**As the reviewer pointed out, we also feel that it is difficult to separate relative contribution of pulsed and global ERK activations to the observed acceleration of cell elimination. From our quick analysis to calculate the ratio of clustered apoptosis to all apoptosis, we did not observe significant differences between early and late phases in control. This is most likely due to the small sample size stemming from low frequencies of apoptosis during the early phase, and even a single occurrence of clustered apoptosis can greatly skew the values. We might be able to see a subtle but significant increase in cluster apoptosis if we could analyze more samples. Even if we could do so, we still may not be able to separate the contribution of ERK pulses and global ERK reduction since the ERK pulse may be caused by reduced ERK activity. Given that we do not currently have a condition where we experimentally negate the ERK pulses without affecting the primary apoptosis, we would not able to make a conclusive statement. We believe that both the basal ERK activity and ERK pulses contribute to maintaining the neighboring LEC survival. To avoid a conclusive statement, we added a new sentence in lines 560-563 (Discussion) “Although the relative contributions of the basal ERK activity and the ERK pulses to the regulation of cluster apoptosis are unclear and still need to be further investigated, one possibility of the role of global downregulation of ERK basal activity is that it may desensitize LECs to the transient ERK pulses and ensure cluster cell elimination occurs during later stages.”.**

Other minor points :
- The text line 86 was a bit missleading : "there is a global EGFR/ERK decrease throughout the LEC elimination period. The dynamics of the decrease in EGFR/ERK parallels with the decrease in endocytic activity in LECs, which was reported in our previous work [14]. While the reduction of endocytic activity ceases around 26 hAPF during the transition into the late phase [14], we observed that EGFR/ERK activity continuously decreases even during the late phase."

It sounds like the authors already showed that there was a reduction of ERK in the LEC cells in ref 14, but this is what is shown here in this paper. Was this piece of text intended to come later in the intro (when summarising the results of this study)?

**Our response**

**We thank the reviewer for careful inspection. We have mistakenly replaced the original text in the introduction. We have now corrected the lines as it was originally as follows:**

**Lines 85-88 “Recent work in our laboratory revealed the network of feedback mechanisms whereby the reduction of endocytic activity promotes both the accumulation of cortical actomyosin and the decrease of junctional E-cadherin, leading to a further decrease in endocytic activity [14]. ”**


Rev. 2:
I very much appreciate the authors' responses to my comments. The authors have addressed many of my comments. However, the authors have not sufficiently addressed my concerns about the characterisation of the clustered cell death and its analysis.

1) In my opinion, a better characterisation of the phenomenon is needed for the reader to fully understand it. (A few of the points have been addressed by the authors in their response to the reviewers, but they should also be presented in the main text of the manuscript.)

a) The position of cell death (as well as of clusters) in the tissue needs to be characterised in more detail. The authors have the tracking data to create maps with the position of cell death in 2D over time. This will help to assess where cell deaths and clusters appear. Do they appear anywhere in the tissue, closer to the histoblasts, or closer to the midline? This is important, as cell death is not randomly distributed in the tissue - more LECs die close to the histoblasts than further away (Nakajima et al., 2011).

**Our response**

**The LECs bordering the histoblasts defined in Nakajima et al., 2011 were located outside of the frame until the transition into the late phase during the live imaging sessions in this study, so we excluded them from the cell tracking and analysis. Based on the current information, the clustered apoptotic events we observed are generally located closer to the midline regions. We now included the description in the text at line 125-127 to clarify the relationship with the previous notions. “Our analysis also did not include the LECs bordering the histoblasts, which are preferentially eliminated around the initiation of histoblast expansion as previously reported [12].”**

b) In Fig. 6E, the authors only show the number of cell deaths but not the percentage of total death. This is shown in Fig. S4C, but it might be better to show this in the main figure as well.

**Our response**

**We have moved Fig. S4C as Fig. 6G.**

c) Are there multiple clusters at the same time?

**Our response**

**There are cases where cluster apoptosis occur at the same time in the two segments, an example of which can be seen in Fig S4B. However, within the same segment, we have not seen multiple concurrent clustered apoptosis.**

d) Do the authors ever classify a cell as belonging to two clusters? This would impact the precision of their analysis.

**Our response**

**We designed the algorithm to define in a way that if a cell is located between two clusters, the two clusters are considered to be a single big cluster. We have added examples of the large clusters in the new Fig. S5A.**

e) A more detailed description of the various shapes of the clusters would be helpful (currently, there is only Fig. S4A). Also, are there certain shapes that are more common?

**Our response**

**We appreciate the interest in more detailed information of the clustered apoptotic events. We have added several representative images of the cluster shapes in Fig. S5A, as well as the sizes of the clustered apoptosis in Fig. S5B. We also have added a few lines to characterize the clustered apoptosis and added references to these new figures in lines 382-385**

**“We observed that the clusters form in lines, clusters, or a combination of lines and clusters containing varying numbers of cells (Fig. S5A). The inhibition of ERK signaling or endocytic activity promoted the occurrences of larger clusters – some reaching more than 10 cells in single clusters (Fig. S5B).”**

2) The authors have not fully addressed my comment on their choice of a 10-minute interval between frames. I was querying whether it is actually okay to argue that cells die at the same time (as a cluster), when indeed there might be a 10-minute difference between their deaths.

Along the same lines, is nuclear breakdown a good marker to assess timing of the cells' death? How fast are the changes in fluorescence and how sure can the authors be that cells with a similar fluorescence are indeed at the same stage of 'their death'? One way to test this could be to do the cluster analysis with a caspase reporter.

**Our response**

**We understand the concerns for our definition of the cell death using the nuclear breakdown as a marker in our study. Our decision initially came from the tracking data from TrackMate which can only detect clear nuclear spots until their degradation. To validate the timing of nuclear breakdown events, we observed both caspase reporter with the nuclear reporter and analyzed the time course from the caspase activation until the end of nuclear breakdown in the new Fig. S2E-F. We saw that nuclear breakdown generally initiates around the final 10 minutes prior to the dispersal of cytoplasmic caspase reporter signals and occurs concurrently with the plasma membrane breakdown/compartmentalization. In addition to the relative short time course, its clear visibility and unambiguity make the nuclear breakdown a good marker for apoptosis. By defining the timing of the end of cell death in this way, it is not appropriate to separate multiple cell death occurring in the same 10-minute time window based on this definition. To reflect this definition in the text, we stated in lines 282-284 “We identified apoptotic LECs using nuclear breakdown as a signature for the end of apoptosis and measured the EGFR activity of LECs in the local vicinity of the apoptotic LECs (Fig. 4E, S2E-F).”. In addition, we consider that clustered apoptosis occurs within a margin of 10-minute intervals. Therefore, we modified a statement to reflect this consideration in the Materials and Methods for cluster detection in lines 689-690 “The loss of cell pairs at the same time or +1 frame (10-minute interval as a margin for nuclear breakdown) was regarded as simultaneous cell death.”**

3) I am not sure whether the authors' simulation of a random distribution of cell death is sufficient to establish that clustering is not due to a mere increase in cell death rate. The reason is that cell death is not randomly distributed in the tissue (there are more cell deaths closer to the histoblasts (Nakajima et al., 2011)). What happens when the authors consider this in their simulations?

Also, as the authors analyse two segments, did they ensure that, when simulating cell removal, they treated the two segments independently? Otherwise, they would have a scenario where they removed one cell from one segment and one from the other, which would never result in neighbouring cells dying. This would skew the analysis. Moreover, it might be good to state the cell death rates used for the simulation in the methods.

**Our response**

**As shown below, our analysis of spatial distribution of cell elimination showed that there is no spatial difference between the regions (lateral vs medial). This is because our analysis did not include the LECs in the regions bordering histoblast nests as we mentioned in the response to the comment in 1a. Therefore, it is unlikely that clustered cell deaths are the consequence of local increase in frequency of single cell deaths derived from differential spatial distribution of cell deaths.**

N.S

Midline vs Lateral: N.S

**Response Fig. 2. Top left. Observable dorsal control LECs split into two equal populations, midline and lateral LECs based on the relative distance to the midline. Bottom Left. Normalized cumulative LEC apoptosis over time for the midline and lateral LECs. Kolmogorov-Smirnov test, N.S. not significant. Bottom Right. Ratio of cells in clustered apoptosis to all apoptosis in each specified region. Student’s T-test, N.S. not significant. N=3 pupae.**

**For the second point, the cell death rates used for the simulation have already been explained in the Materials and Methods section as in Lines 697-699 “The initial positions of the cells are the XY positions at the first frame, and (2) cell death rate follows the experimental rates. All samples of the associated genotypes from the experiments were used as inputs for the simulation, each iterated for random cell removals for six times.”**

4) Although I understand the authors' concerns about including cell outline data in this manuscript, I think that the description of cluster apoptosis is linked with the issue of tissue integrity and that this needs to be addressed in this manuscript. Thus, it would be important to include cell outline data showing how clustered apoptosis impacts on cell removal and tissue integrity.
In addition, looking at cell outlines will help to establish neighbourhoods and improve the characterisation of the phenomenon (see comment 1).
The analysis should be more detailed than what the authors have provided in the figure in the response to the reviewers and include an assessment of how many neighbouring cells delaminate at (almost) the same time and how this relates to clustered apoptosis.

**Our response**

**We appreciate the comment on the discussion about epithelial sealing following clustered apoptosis. Our analysis of the cell outlines showed that there does not seem to be any impairment in epithelial sealing upon clustered apoptosis, which is shown in our previous response and the new Fig. S5C. We assume that this is due to the fluid character of this tissue with the constant movements of both LECs and histoblasts, especially during the elimination period. The areas where clustered apoptosis occur appear to be immediately covered by the surrounding cells migrating toward the midline. We have added an additional sentence for this idea in lines 543-547 “In fact, fluorescent labeling of cell boundaries showed that there is no visible impairment in epithelial sealing despite the presence of clustered apoptosis (Fig. S5C). This observation contrasts with the formation of transient gaps between cells when cell deaths were artificially induced in the Drosophila pupal thorax [30]. The constant movement of both LECs and histoblasts may prevent the formation of transient gaps in the abdominal epidermis. ”**

5) When discussing ERK activity, the authors consider one cell with its six direct neighbours (scheme in Fig. 4E). How can this lead to the formation of clusters in the shape of a line of four cells?

**Our study mainly focused on the modulation of ERK activity which determines the presence of adjacent cells undergoing apoptosis. Without ERK activity reaching the required thresholds reached by the basal ERK activity or ERK pulses to maintain cell survival, there seems to be a default mechanism to locally induce multiple cell deaths. It is interesting that there is a mechanism by which neighboring cells die simultaneously in the absence of the ERK pulses. Although this is out of scope of this manuscript, we speculate that the death can sometimes propagate in a way that secondary apoptotic events act as a primary apoptosis to induce the cell death of the cell next to it. If this happens in line, apoptotic clusters of four cells in a line could be formed.**

Other comments:
1) I am very sorry that I did not spot this in my first review, but I have noticed that, using pnr-Gal4, it appears that not all LECs are labelled. How do the unlabelled cells behave? At least, it should be stated in the text that not all LECs were considered.

**Our response
*pnr*-expressing LECs are the majority of cells located in the middle of the segments, but not at the segment boundary as shown in Fig. 1A. We have added further clarification in our description of LECs in Lines 123-125 “we either genetically knocked-down or overexpressed EGFR in the majority of LECs under the control of *pnr*-GAL4, except for LECs near the segmental boundary (Fig. 1A).”**

2) I would like to thank the authors for clarifying the point about the data from 18ºC and 25ºC (Fig. 6L). I think that the temperature dependence should be mentioned in the main text.

**Our response**

**We have added a sentence in the results section to mention the effects of temperature shift in Lines 403-406 “We note that there is a minor increase of clustered elimination in the 18-29 ºC shifted control LECs compared to the 25 ºC LECs, which we consider to be caused by the minor increase on the cell elimination rate at higher incubation temperatures (Fig. 1E, H).”**

Rev. 3:
The authors have adequately responded to most of my comments. I have only a minor suggestion.

Regarding their response to comment #1:
I understand the challenges of tracking the ratio of nuclear to cytoplasmic miniCic intensity. Alternatively, I would suggest using TrackMate to measure the nuclear miniCic intensity for a few data in Figures 2, 3, and 6, for instance Fig. 2F control, and qualitatively cross-check whether the tendency observed from the ratio is consistent with the one using the nuclear intensity. Plotting such data side by side could be a helpful supplemental data.

Otherwise, I congratulate the authors on this intriguing study.

**Our response**

**We are glad to hear the kind words. We have added a representative nuclear miniCic intensity for the Fig. 2F control as Fig. S1E, which shows the same general trend of increase in miniCic over time.**
